# The intrinsic dimension of gene expression during cell differentiation

**DOI:** 10.1093/nar/gkaf805

**Published:** 2025-08-30

**Authors:** Marta Biondo, Niccolò Cirone, Filippo Valle, Silvia Lazzardi, Michele Caselle, Matteo Osella

**Affiliations:** Department of Physics, University of Turin and INFN, via P. Giuria 1, I-10125 Turin, Italy; Department of Physics, University of Turin and INFN, via P. Giuria 1, I-10125 Turin, Italy; Department of Physics, University of Turin and INFN, via P. Giuria 1, I-10125 Turin, Italy; Department of Physics, University of Turin and INFN, via P. Giuria 1, I-10125 Turin, Italy; Department of Physics, University of Turin and INFN, via P. Giuria 1, I-10125 Turin, Italy; Department of Physics, University of Turin and INFN, via P. Giuria 1, I-10125 Turin, Italy

## Abstract

Waddington’s epigenetic landscape has long served as a conceptual framework for understanding cell fate decisions. The landscape’s geometry encodes the molecular mechanisms that guide the gene expression profiles of uncommitted cells toward terminally differentiated cell types. In this study, we demonstrate that applying the concept of intrinsic dimension to single-cell transcriptomic data can effectively capture trends in expression trajectories, supporting this framework. This approach allows us to define a robust cell potency score without relying on prior biological information. By analyzing an extensive collection of datasets from various species, experimental protocols, and differentiation processes, we validate our method and successfully reproduce established hierarchies of cell type potency. Our work provides a direct link between geometric properties of single-cell expression profiles and the level of differentiation of a cell population.

## Graphical Abstract

**Graphical Abstract:**
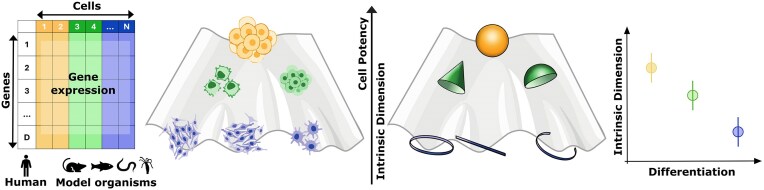

## Introduction

The Waddington’s epigenetic landscape is often used as a metaphor to rationalize the cell differentiation process [1]. Uncommitted progenitor cells progressively lose their potency as they roll down on a high-dimensional surface whose geometry reflects the biological constraints and complex regulations that canalize the differentiation trajectories. Finally, the cells end up in stable basins representing terminally differentiated cell types. Since a cell state is essentially defined by its gene expression profile, expression levels are the natural coordinates in this large epigenetic space [2]. These coordinates, or at least a proxy, can be experimentally measured thanks to recent innovations in single-cell RNA-sequencing (scRNA-seq) [3, 4].

While Waddington’s evocative description has been instrumental in interpreting several results in developmental biology [5], the extent to which this picture is supported by large-scale empirical data remains to be tested. The trajectories followed by single cells in the expression space during differentiation should reflect the presence of an underlying landscape and its specific geometry. However, how to confirm this intuition using RNA sequencing data, how the information about the landscape geometry can be extracted, and how it is related to the differentiation potential are still largely open questions.

Rugged and high-dimensional landscapes, as the one depicted in Waddington’s drawings, are ubiquitous in statistical physics. They typically represent energy surfaces of complex systems such as spin glasses [6, 7], but analogous descriptions are used for fitness landscapes in evolutionary biology [8] or for loss functions of artificial neural networks in machine learning [9]. We plan to leverage on these analogies to identify geometry-based observables that can reveal the presence of an underlying landscape guiding differentiation from scRNA-seq data. Cell positions on the landscape can then be used to define a potency score only relying on data geometry, without the need for prior biological knowledge or well defined “marker genes,” whose expression is often used to annotate cell types [10].

More specifically, the analogy with statistical physics systems suggests that we can consider pluripotent cells as cells in a ‘high-temperature” state. In fact, they should be able to freely navigate the landscape, only to become progressively constrained into a specific valley when they commit to a differentiation path. Differentiation would correspond to a “freezing process,” during which the expression profiles are narrowed in the manifolds of cell types. This picture agrees with the observation that multipotent cells do not typically have a specific and conserved expression profile or clear-cut markers [11]. Stemness seems rather characterized by pervasive transcription [12] and by a general high level of heterogeneity [13, 14].

This paper will test the presence of a trend that captures the progressive reduction of the accessible expression space during differentiation using the concept of intrinsic dimension. Data associated with complex systems are often high-dimensional, yet statistical regularities stemming from dependencies and correlations tend to concentrate data points on low-dimensional manifolds [15]. Also gene expression data, and particularly RNA-sequencing data, exhibit various statistical laws and correlation patterns [16, 17], suggesting that they can be efficiently described by a number of variables significantly smaller than the large gene repertoire. This notion has been previously suggested [18, 19], and it is implicitly assumed by most data pre-processing pipelines that incorporate dimensionality reduction steps [20].

The hypothesis this paper focuses on is that the intrinsic dimension of cell expression profiles decreases with cell differentiation, and thus this quantity can be used to robustly measure the potency of a cell population. Drawing an analogy between the Waddington’s landscape and the energy profile of statistical physics models, such as the Hopfield model, we confirm this intuition by simulating “differentiation” through a reduction of the system’s temperature. Subsequently, we develop a potency score based on the intrinsic dimension of expression profiles and confirm its efficacy in capturing differentiation processes, and in distinguishing stem or pluripotent cells from committed cells across various species and biological contexts.

## Materials and methods

### Datasets

We collected recently published scRNA-seq datasets that are readily accessible through the GEO repository [21] or other online repositories. These datasets span various model organisms, and describe embryonic development, organ/tissue development or specific differentiation lineages. The experiments were performed in independent laboratories with different protocols. However, we only selected experiments using Unique Molecular Identifiers (UMIs), with the exception of the dataset on human gastrulation [22] . The full list of datasets is reported in Supplementary Table S1 and briefly described in the Supplementary Information File.

### Data pre-processing

Cells were filtered according to three criteria based on thresholds on the total number of reads, the number of detected genes and the mitochondrial percent. We directly applied to each dataset the thresholds reported by the authors (Supplementary Information File, Section 1). When applicable, doublets identified by the authors were also excluded.

We only considered protein-coding genes as annotated by the data mining tool BioMart, accessible via the Ensembl database [23]. A complete list of protein-coding genes is not available for *Hydra vulgaris*. Therefore, in this case, we used all the genes contained in the dataset.

A relevant step in most analysis pipelines of scRNAseq data is the normalization introduced to partially compensates for heterogeneity in sequencing depth [10]. We simply divided the gene transcript counts in a cell by the total number of detected transcripts, defining cell transcriptional profiles with relative abundances.

### The intrinsic dimension of single-cell RNA sequencing data and how to use it

The output of a scRNA-seq experiment is a count matrix reporting the number of detected transcripts from the *D* possible genes in the *N* single cells that have been sequenced. This count matrix naturally defines a set of *N* points in a *D*-dimensional expression space. However, as discussed in the Introduction, we do not expect that these points can randomly occupy the whole expression space due to regulatory mechanisms and constraints. Therefore, the system should display an intrinsic dimension (ID) lower than the embedding dimension *D*. The ID represents the number of coordinates actually needed to approximately specify the positions of data points with minimal information loss [24]. From a geometric perspective, the data points of several complex systems belong to a relatively low dimensional manifold embedded in the high dimensional data space [15]. In these cases, the ID precisely represents the dimension of the data manifold. The problem of estimating the ID of a dataset has been faced in multiple fields, from physics to computer science, and several ID estimators have been proposed [25–29]. We considered different estimators and analyzed their strengths and weaknesses in the context of expression data. This section summarizes how a robust ID-score can be defined and evaluated on different scRNA-seq dataset (a more detailed description of the estimators is provided in the Supplementary Information File, Sections 1 and 2).

We selected algorithms belonging to two main categories: projective and geometric estimators. Projective methods are essentially based on the analysis of the eigenvalues of the *D* × *D* data covariance matrix and aim to extract the minimal number of directions that captures the data variance [28, 29]. Principal component analysis (PCA) is an example of a popular linear projective method. On the other hand, geometric or fractal methods leverage on the observation that the density of neighboring points at a fixed distance depends on the ID of the data manifold [25–27].

All ID estimators depend on the sample size, defined by the number of sequenced cells, at least when the system is undersampled (Supplementary Information File, Section 1). Given the high dimensionality of the expression space compared to the typical number of sequenced cells (often one or two orders of magnitude less), gene expression datasets are reasonably in the under-sampled regime. In fact, estimators do not converge on the scRNA-seq datasets we analyzed (Supplementary Fig. S1). The convergence behavior of different estimators with the sample size remains poorly characterized, therefore it is not trivial to extrapolate the correct ID.

To address this issue, we compare the estimated IDs of different cellular populations using the same sample size (75% of the least represented population) through 10 random sub-samplings. This method also provides a measure of uncertainty, shown as error bars in the figures, consisting in the standard deviation of ID across sub-samples. As expected for a complex and structured system, the absolute ID values we measure are significantly smaller than the gene repertoire, ranging from units to hundreds depending on the cell populations we consider (Supplementary Table S1). These values still depend on the sub-sampling size. However, we are mainly interested in relative values and trends, such as the relative potency of cell sub-populations, the cell potency as a function of time or differentiation stage. Therefore, we can define an ID-score by rescaling the ID measurements in the [0, 1] range (Supplementary Equation S1).

As mentioned before, we tested the robustness of the results across ID estimators (Supplementary Figs S13–S16), but in the main body of this paper we adopt an ID-score based on a specific geometric estimator, called TWO-NN [26, 30]. This estimator is based on the statistics of the distances between each point and its first two neighbors. The focus on such a local property of the dataset makes it more robust to curvature effects and less sensible to outliers with respect to other methods such as PCA. The detailed comparative analysis of different estimators is reported in Supplementary Information File, Section 2.

Before analyzing actual biological datasets, we used a toy model to test the basic hypothesis that a system defined by a rugged landscape displays a decrease in ID as the trajectories are progressively constrained by the landscape geometry. We confirmed our hypothesis using the Hopfield model [6] as a testing ground (the detailed analysis is reported in Supplementary Information File, Section 3). Although this model was originally developed for memory storage in the brain [31], it has often been used as a possible abstract mathematical formalization of the Waddington’s landscape [32–35]. We mimicked the differentiation process by progressively freezing the system. The trend of the ID of configurations with temperature confirms our initial intuition (Supplementary Fig. S2).

## Results

### The intrinsic dimension decreases with developmental time

**Figure 1.**
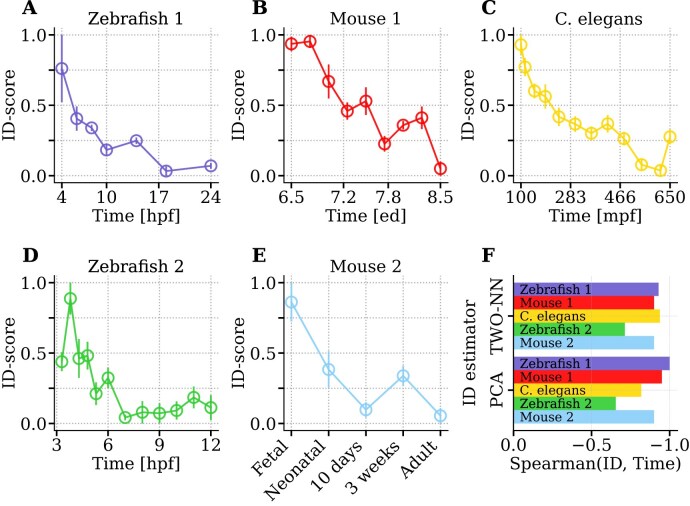
The contraction of the intrinsic dimension of expression profiles during embryo development. ID measurements performed over 10 sub-samplings and re-scaled in the [0; 1] interval (see the “Materials and methods” section) are reported (circle ± error bar = mean ± standard deviation). Supplementary Table S1 specifies the number of cells and genes considered for each dataset. The different panels refer to (**A**) Zebrafish embryogenesis [36], spanning from 4 to 24 h post-fertilization (hpf); (**B**) Mouse gastrulation and early organogenesis [37] between embryonic day (ed) 6.5 and 8.5; (**C**) *Caenorhabditis elegans* embryogenesis [38] from 100 to 650 min post-fertilization (mpf); (**D**) Zebrafish embryogenesis [39], in the time window 3.3–12 hpf; (**E**) Mouse Cell Atlas [40, 41], fetal to adult progression. (**F**) Spearman correlation between developmental time and the ID calculated with TWO-NN and a PCA-based estimator, specified in Supplementary Equation S5.

As a first test of a link between differentiation and ID, we analyzed a large number of datasets related to embryonic and fetal development across various model organisms. The global level of specialization in the cell population generally increases with developmental time, and this should induce a reduction of the ID. Indeed, Fig. 1 shows a robust and consistent decreasing trend during embryo development of different species (*Caenorhabditis elegans*, mouse, and zebrafish). The trend spans several developmental time windows and looks progressive, rather than characterized by an abrupt transition at a specific point. In fact, we can observe a decrease in the intrinsic dimension during the first days of mouse gastrulation (Fig. 1B) but also comparing fetal versus neonatal or adult cells (Fig. 1E). Datasets from independent studies on the same biological system, based on different experimental and sequencing protocols (as in the case of Fig. 1A and D), lead to analogous conclusions, thus supporting the robustness of the trend.

As motivated in the previous section, the ID-score reported in the figures is based on the TWO-NN estimator. However, the ID dependence on developmental time does not depend on this choice. As an illustrative example, we can consider two estimators based on very different assumptions and on different scales. Specifically, we can compare the local geometric estimator TWO-NN [26] with PCA-based observables (such as the methods described by Supplementary Equations S5–S7 in Section 2 of Supplementary Information File) that focus on the diversity of the eigenvalues of the whole dataset covariance matrix. Figure 1F shows that one of these alternative ID quantification indeed leads to similar temporal trends across all different datasets, as confirmed by the high values of the Spearman coefficient. The more comprehensive analysis reported in Section 2 of the Supplementary Information File proves that the agreement between estimators is more general. However, methods based on “local” statistical properties, such as TWO-NN, are less affected by the presence of small sub-populations of cells with radically different profiles, which can strongly affect the measured ID when using projective methods. An example, which we discuss in Supplementary Fig. S3C and D, is the presence of mature red blood cells in the sample. Indeed, these enucleated cells present a highly specific expression profile [42].

**Figure 2.**
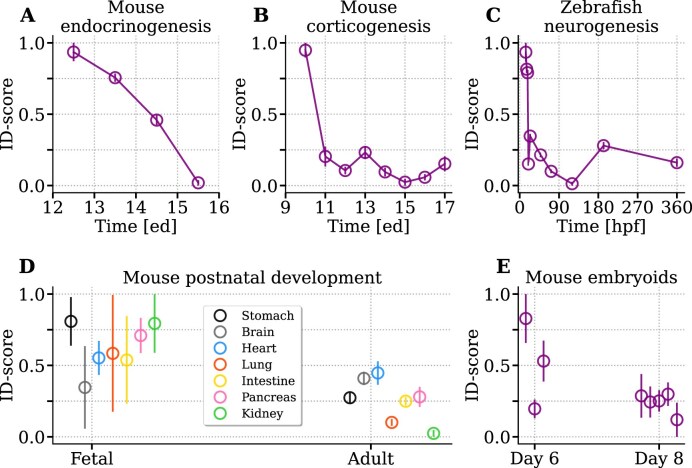
Organogenesis is accompanied by a decrease of expression intrinsic dimension. We analyzed the following organ developmental processes: (**A**) Mouse pancreatic endocrinogenes between 12.5 and 15.5 ed [44]; (**B**) mouse corticogenesis [45] in the time window 10.5–17.5 ed; (**C**) Zebrafish neurogenesis [46] between 14 and 360 hpf. (**D**) The Mouse Cell Atlas dataset [40, 41] allows the comparison between the fetal and adult stage of 7 organs. (**E**) Finally, embryoid development [43] is analyzed. Three biological samples of embryods cultured for 6 days are compared to five embryoids cultured for 8 days.

The decreasing trend in the ID-score is not a sole property of the whole embryo. It can be equally observed by examining the development of a single organ or tissue, as depicted in Fig. 2. This observation suggests a form of scale-invariance with respect to anatomical resolution. In particular, Fig. 2A and B focus respectively on pancreatic endocrinogenesis and on the formation of the cerebral cortex in mouse. Figure 2C refers to zebrafish neurogenesis. Figure 2D provides instead a comparison between fetal and adult stages of seven organs. Despite the significant variability between organs, a consistent decrease in ID after development can be observed. The same behavior emerges when considering artificial embryoids [43] (Fig. 2E). The expression profiles of mouse embryoids at day 8, which completed gastrulation to neurulation and organogenesis, have a clearly lower ID with respect to the less differentiated embryoids at day 6.

In a Waddington’s landscape scenario, the reduction of the ID should be generally accompanied by an increase in the correlation between genes expression levels, as a result of the geometrical constraints due to gene regulation. As a large-scale measure of the level of correlation, we can consider the size of the gene–gene network whose links represent linear correlations above a certain threshold. The network size grows with differentiation (Supplementary Fig. S4), indicating a global increase of correlation. An analogous trend can be observed by changing the temperature in the Hopfield model.

The trends reported in Figs 1 and 2, although overall consistent, exhibit noticeable fluctuations, in some cases exceeding the variability estimated through random subsampling (see “Materials and methods” section). It is important to recognize that the differentiation of an entire embryo or tissue involves the asynchronous emergence of multiple cell types, each potentially exhibiting different levels of specialization, within an inherently stochastic process [47–49]. Additional variability may also arise from technical factors such as batch effects or the relatively small number of cells recovered for sequencing. While the uncertainty in the ID-score estimated via random subsampling reflects fluctuations due to limited sampling, it could not fully capture the true extent of ID variability for heterogeneous populations.

### The intrinsic dimension reflects the differentiation potential

The observed decrease of the ID with developmental time can be ascribed to two main possible factors: the overall progressive differentiation of the cell population and the proliferation in the number of the cell types during tissue and organ formation. There is indeed a possible relation linking the measured ID and the number of cell types, which typically grows during embryogenesis (Supplementary Fig. S5).

In a Waddington’s landscape scenario, cell types are represented by different basins of attraction that can have specific geometries and intrinsic dimensionality, depending on the level of complexity and the degree of gene regulation defining each cell type. Therefore, the expression profiles of cells composing an organ or a whole embryo are expected to lie on a structured and composite manifold. ID estimators are affected by this heterogeneity. Specifically, as we show in detail in Section 2 of the Supplementary Information File, when the data points belong to a composition of manifolds with heterogeneous dimensions, the ID estimate given by TWO-NN is dominated by the low-dimensional manifolds. Therefore, the simple increase in the number of cell types, which typically occurs during development, can induce a trend in ID. In fact, there is an increasing chance of observing a cell type associated to a low dimensional manifold if their number increases and they have heterogeneous IDs (Supplementary Figs S6 and S7).

Since we are interested in evaluating the ID as a score for cell potency, we need to disentangle this spurious effect and quantify the actual correlation between ID and differentiation level. To this aim, we collected several well established and annotated differentiation trajectories. Along these trajectories, cell types can be roughly ordered by their differentiation level, and we can test if the ID can recapitulate this ordering without relying on prior biological information.

**Figure 3.**
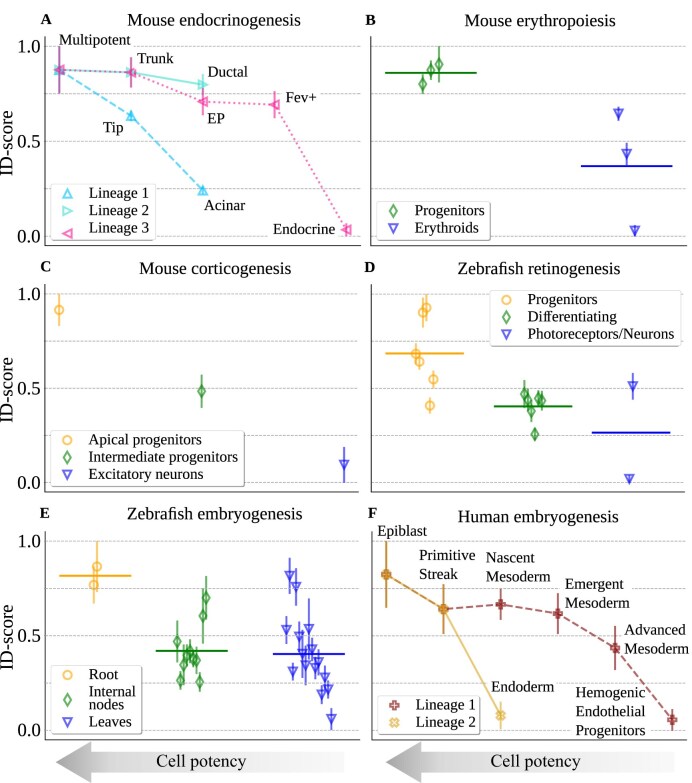
The intrinsic dimension reflects the potency of cell types along developmental lineages. (**A**) The hierarchy of cell types along three main differentiation lineages of pancreatic endocrinogenesis (as reconstructed in [44]) can be well reproduced by the intrinsic dimension. (**B**) In the production of mouse erythroids [37], the ID-score can distinguish the class of haematoendothelial and blood progenitors from erythroids. Horizontal lines show the average ID-score of cell types belonging to a similar potency level. (**C**) In mouse corticogenesis [45], we can correctly order apical progenitors, intermediate progenitors and excitatory neurons. (**D**) In the formation of retinal neurons in zebrafish [50], different cell types can be group in the broad classes of retinal progenitors, differentiating retinal cells and retinal neurons/photoreceptors. The ID-score can well separate their different levels of differentiation. The specific cell types are reported in Supplementary Information File, Section 8. (**E**) Cell types from zebrafish embryos are roughly ordered according to their position in the developmental tree reconstructed in [39]. (**F**) The hierarchy of cell types along two differentiation lineages of human gastrulation (as reconstructed in [22]) is reflected by the ID-score.

As a first example, the process of pancreatic endocrinogenesis in mouse is known in sufficient detail to draw the diagram of the lineage relationships between pancreatic cells that summarizes the differentiation process [44]. Figure 3A shows that the ID-score can correctly order the cell types in terms of their potency along the differentiation lineages, correctly reproducing the known relationships between cell types only from data geometry.

The developmental lineage relative to erythroid cell formation during mouse gastrulation has also been reconstructed in detail [37]. In particular, cell types can be ordered according to their potency from progenitor cells to the final erythroids. Figure 3B demonstrate that the ID can clearly separate the cell types belonging to these two classes. Similarly, the apical and intermediate progenitors can be compared to excitatory neurons, a fully differentiated cell type, in the process of mouse corticogenesis [45]. Also in this case, the ID-score shows that the progressive differentiation corresponds to a reduction of the dimensionality (Fig. 3C).

An analogous analysis can be performed on different species to test the robustness of the results. Zebrafish embryogenesis is a well studied system in which the cell types have been characterized and can be ordered by potency along differentiation lineages. In particular, looking at the expression of well-established gene markers, Farnsworth et al. annotated three main cell clusters with increasing level of specialization: retinal progenitors, differentiating retinal cells and final retinal neurons and photoreceptors [50]. Figure 3D shows how the ID correctly reproduces this potency ranking.

Finally, in reference [39], an alternative diffusion-based computational framework was used to infer the developmental trajectories in zebrafish embryogenesis. Each cell type can thus be placed on a tree-like structure. The tree root, corresponding to pluripotent cells, and the different branch annotations were validated by marker gene expression patterns. Starting from these inferred lineages, we can distinguish three groups of cell types of decreasing potency level: the cell types close to the tree root, the intermediate cell types along the branches, and finally the differentiated cell types at the tree leaves. Figure 3E reports the coherent decrease of the ID along the differentiation tree.

To further assess the applicability and robustness of the ID-score as a measure of cell potency, we evaluated its performance on human data. In particular, reference [22] provides scRNA-seq data from a human gastrula at 16–19 days post-fertilization. In that study, the authors identified the epiblast cluster and reconstructed two major developmental trajectories originating from the epiblast and the primitive streak using diffusion maps and RNA velocity analysis. One trajectory leads to the endoderm, while another progresses through nascent, emergent, and advanced mesodermal states, eventually giving rise to hemato-endothelial progenitors. Figure 3F shows that, also in this context, the ID-score captures the decline in cell potency along both developmental paths. Although this analysis only considers well-populated cell types for statistical robustness, Supplementary Fig. S8 confirms that the observed trends persist when the ID-score is applied across the full dataset.

The ID seems to recapitulate the potency trends in dynamic differentiation processes related to embryo development and organogenesis. However, a general measure of potency should be able to discriminate between pluripotent and differentiated cells also during tissue homeostasis, maintenance, and regeneration. As a first test of this broader applicability, we consider the well-established model organism *Hydra vulgaris*. This small freshwater polyp has been highly studied due to its exceptional regenerative capacity and continuous tissue renewal. In fact, unlike most model organisms, Hydra maintains active stem cell populations throughout its life, enabling both asexual reproduction and whole-body regeneration [53–57].

**Figure 4.**
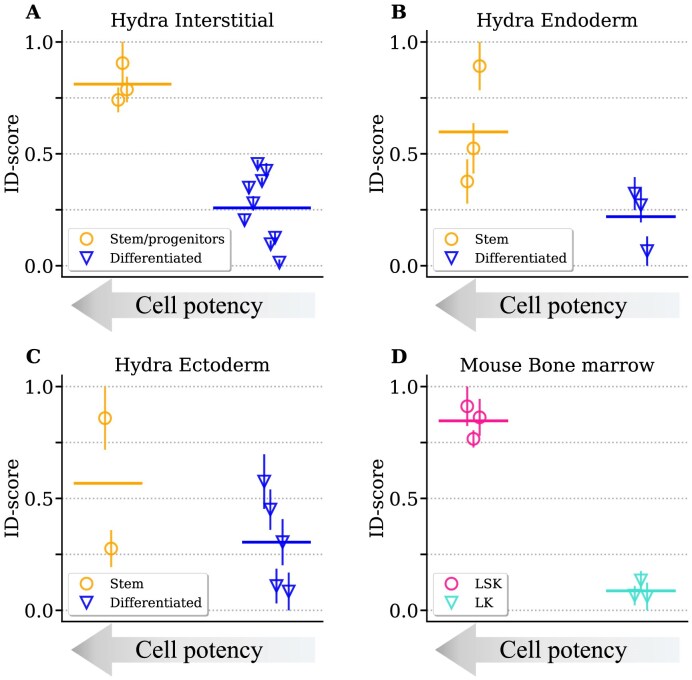
The intrinsic dimension can discriminate between stem cells and differentiated cells in tissue turnover. The ID-scores relative to clusters of stem/progenitor cells versus differentiated cells are compared for the three hydra epithelial layers: interstitial (**A**) endoderm (**B**) and ectoderm (**C**) (data from [51]). The specific cell types corresponding to each point are reported in Supplementary Information File, Section 8. (**D**) The ID-score of two cellular populations (LSK, LK) isolated in hematopoietic stem cells [52] is reported.

The simple body plan of this organism has allowed the identification of three main independent stem lineages—ectodermal epithelial, endodermal epithelial, and interstitial stem cells—which remain active throughout the animal’s life [58]. Single-cell expression profiles of stem cells have been isolated in all three lineages using a combination of computational tools and prior knowledge based on marker genes and spatial localization [51]. The ID of genome-wide expression profiles can clearly capture the difference in potency between stem and specialized cell types in all three lineages (Fig. 4A–C). The separation between the ID-scores of stem/progenitor and differentiated cells is more pronounced for the interstitial lineage (Fig. 4A), with respect to the epithelial ones (Fig. 4B and C), suggesting a more drastic drop in potency. In fact, epithelial stem cells in the ectoderm and endoderm are highly proliferative, but exhibit limited differentiation potential with respect to interstitial stem cells, which can produce a broad range of specialized cells [53]. This difference is also reflected in the intrinsic dimensions values that we observe before the [0, 1] scaling: a wide range of values for cell clusters in the interstitial lineages with respect to the values of the epithelial lineages (Supplementary Table S1).

As a final example, we consider hematopoiesis, i.e. the process of continual turnover of blood cells driven by hematopoietic stem cells in the bone marrow. A large-scale study in mouse identified two sub-populations (LSK and LK) with a different level of maturity in hematopoietic stem and progenitor cells using a sorting procedure based on two markers [52]. LSK cells are early progenitors, not committed to a specific blood cell lineage, and express high values of Sca-1 (stem cell antigen-1) and c-Kit (a transmembrane receptor associated to immature state). On the other hand, LK cells exhibit low levels of c-Kit, indicating an intermediate state of maturity. Once again, the ID can well discriminate the different differentiation potentials of the two sub-populations (Fig. 4D).

## Discussion

The process of cell differentiation is a comprehensive reorganization of cellular physiology. The robust and consistent differentiation patterns observed at the macroscopic level are ultimately determined by a complex orchestration of a high number of molecular processes heavily subjected to stochasticity at the single-cell level.

In analogy to statistical physics systems, an ordered macroscopic state emerges from a high-dimensional and noisy microscopic behavior [59]. Therefore, even if key specific regulators play a dominant role in differentiation [60–62], large-scale observables should more reliably capture the extensive molecular reprogramming involved in differentiation with respect to the inherently noisy expression of a handful of “marker genes” [63]. Modern scRNA-seq techniques offer an access to such genome-wide observables.

Taking inspiration from the Waddington’s landscape picture, we have shown that indeed global geometrical properties of single-cell expression profiles are indicative of the differentiation level, which can be robustly recapitulated by a single score based on the intrinsic dimension. Importantly, this ID-score does not require specific or complex data pre-processing or prior biological knowledge about the system. For instance, the reported ID trends are robust to common gene selection techniques (Supplementary Fig. S9) and consistently emerge across species and tissues. This simplicity and robustness of the ID-score allows its straightforward integration in current analysis pipelines [10]. If different sub-populations are identified in a sample, for example through clustering or thanks to known relevant molecular players, the ID-score can order these cell groups along a potency line.

A plethora of computational methods, often called pseudotime inference tools, try to align single cells along trajectories using the similarity of their expression profiles [39, 64–67]. These trajectories should reflect continuous biological processes such as developmental paths, although inferred from static expression snapshots. However, the vast majority of these methods need prior information about the identity of the starting (or end) cells, thus ultimately about the differentiation direction [64]. Our ID-score can robustly provide the correct ordering of cell groups or equivalently the pseudotime direction, thus constraining the likely differentiation diagrams.

**Figure 5.**
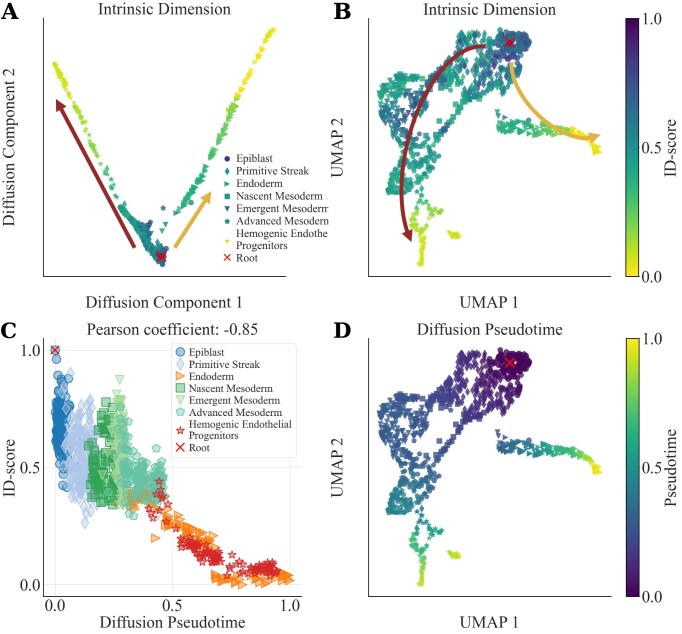
The ID-score as a support for trajectory inference and pseudotime analysis. (**A**) We computed the diffusion map of selected cell types from the human gastrulation dataset [22]. Cells are colored with the ID-score calculated using their 70 nearest neighbors in the two-dimensional embedding. Arrows highlight the mesoderm (brown) and endoderm (gold) branches. The putative root cell (red cross) is the cell with the highest ID-score. Colors in the legend correspond to the average ID-score of each cell types and consistently reflect the cell type potency hierarchy, in agreement with the result in Fig. 3F. UMAP plots of expression profiles using 2000 highly variable genes are displayed in (**B**) and (**D**). Cells are color-coded using the ID-score (B) or the diffusion pseudotime (D). (**C)** The scatterplot illustrates the strong negative correlation (Pearson coefficient = −0.85) between the ID-score and the diffusion pseudotime using the same root cell. A detailed description of the analysis is reported in Supplementary Information File, Section 6.

We can better illustrate this point using the human gastrulation dataset of [22] as an example. Transcriptional profiles of single cells are embedded into a low-dimensional space using diffusion maps, as in Fig. 5A. However, to reconstruct developmental trajectories and perform pseudotime analysis, one must select a “root” cell [68, 69]. The ID-score can be naturally applied for this task by computing a “local” ID value for each cell based on its neighbors in the diffusion space. Cells are colored by this ID-score in Fig. 5A, and a similar coloring can be applied to a Uniform Manifold Approximation and Projection (UMAP) embedding [10] (Fig. 5B). Notably, cells with the highest ID-scores are located within the epiblast, the pluripotent population at the origin of major differentiation pathways. The cell with the maximum ID-score can thus be selected as the root for trajectory inference, yielding a developmental map that aligns closely with known lineage progression. Using the same root cell, we can compute the diffusion pseudotime, which shows a strong correlation with the ID-score (Fig. 5C). Indeed, the UMAP representations colored by pseudotime and by ID-score are visually similar (Fig. 5B and D), reinforcing the coherence between these two approaches. While we focus here on a subset of well-represented cell types (i.e., with >90 cells), the full detailed analysis—performed on the entire dataset with similar results—is available in Supplementary Fig. S8. In conclusion, the ID-score can support established methods of trajectory inference and offers a complementary, data-driven criterion for root cell selection and pseudotime validation. This is particularly valuable in developmental systems or organisms where cell type annotation remains incomplete or uncertain.

More generally, time clearly plays a crucial role in development. However, the order and tempos of differentiation steps are not conserved across species and systems [70]. Therefore, the relationship between time and differentiation potential could be arbitrarily complex and system-dependent. As RNA sequencing becomes more and more precise, our quantitative measure of potency can be used to estimate the actual rate of differentiation over time, unlocking the possibility of quantitative comparisons, for example of the speed of embryo or tissue development across different species [71] or between natural and laboratory models such as organoids [43, 72].

The analogy between the Waddington’s landscape and statistical physics systems has long been recognized. Consequently, tools and ideas from statistical mechanics have percolated into single-cell analysis [14, 73]. Specifically, measures based on the entropy of expression profiles have been proposed as proxies for stemness [74–79]. The basic idea stems from the observation that high variability in expression profiles is often associated with stem cells, and entropy is a theoretically grounded measure of variability that goes beyond simple variance-based evaluations [74]. While entropy seems to capture known differentiation trends in some datasets, it fails in several instances where the ID-score robustly recovers known potency hierarchies (Supplementary Fig. S12C and F). These two quantities capture different, and in principle, independent aspects of the dataset statistics, and thus can be jointly used to extract global patterns in expression profiles. However, entropy values depend on the number of available states of the system, which is set by the number of detected genes. Unfortunately, the sparsity of scRNA-seq datasets is strongly influenced by technical noise due to the sampling process of RNA sequencing [17]. This effect suggests a higher robustness of geometry-based measures like intrinsic dimension. In fact, to enhance robustness, many proposed entropy-based tools integrate additional information, which may not always be available, such as the protein-protein interaction network [79] or gene functional annotations [77].

Similar considerations hold true for another proposed tool for potency estimation, which leverages directly on the total number of expressed genes [80]. Transcriptional diversity often correlates with potency, probably because differentiated cells selectively switch-off certain pathways. However, this measure does not always capture potency trends in the datasets we explored (Supplementary Fig. S12B and E), possibly due to its high sensitivity to sampling noise.

Cell proliferation is another important correlate of cell potency, as progenitor cells are typically more proliferative than their differentiated progeny, and strong links exist between cell cycle regulation and cell fate decisions [81, 82]. Therefore, there may be a direct or indirect relationship between the intrinsic dimension of the expression profiles and the level of cell proliferation. Several transcriptional signatures associated with proliferation can be derived from gene expression data [82–84]. In the Supplementary Information File, Section 7, we detail the two alternative proxies for cell proliferation that we considered. As expected, these proliferation proxies generally show a negative correlation with the level of differentiation; however, we observe several exceptions (Supplementary Fig. S10). Notably, the ID-score and proliferation measures—such as the percentage of cycling cells—appear to capture distinct aspects of cell identity: while they are often correlated, they can show divergent trends in specific datasets (Supplementary Fig. S11). In these cases, the ID-score more consistently reflects known hierarchies of cell potency. Further studies using direct measurements of cell-cycle phase and cell growth could help clarify the extent to which global geometrical properties of expression profiles are influenced by proliferation dynamics.

Defining a measure that can quantitatively and robustly capture the potency level of a cell population is useful beyond the reconstruction of natural developmental trajectories. For example, cell reprogramming experiments promise to have relevant applications from regenerative medicine to disease modelling and drug testing [61, 85, 86]. In reprogramming protocols, the goal is to induce pluripotency in differentiated cells. A quantitative potency measure, such as the ID-score, can complement existing methods by providing a marker-free metric to identify cell sub-populations that have successfully achieved pluripotency and to assess the extent of their potency.

Finally, the Waddington’s landscape metaphor has also been invoked for understanding cancer etiology and progression [87]. This parallel suggests that our approach could represent a useful quantitative tool in this context as well.

## Supplementary Material

gkaf805_Supplemental_File

## Data Availability

The scRNA-seq datasets we analyzed (Supplementary Information File, Section 8) are publicly accessible through the links provided in the corresponding papers. The original code is available at: https://github.com/BioPhys-Turin/The-intrinsic-dimension-of-gene-expression-during-cell-differentiation and https://doi.org/10.5281/zenodo.16422105. Additional data requests can be directed to the corresponding author (M.O.).
