# Supplementary material for: The intrinsic dimension of gene expression during cell differentiation
Source: Nucleic Acids Res. 2025 Aug 30;53(16):gkaf805. doi: 10.1093/nar/gkaf805 (PMC12401001; doi:10.1093/nar/gkaf805)
Supplement: gkaf805_Supplemental_File [file gkaf805_supplemental_file.pdf]

Supplementary Information for: The intrinsic dimension of gene expression during cell differentiation

Marta Biondo, Niccolò Cirone, Filippo Valle, Silvia Lazzardi, Michele Caselle, Matteo Osella

Contents

1 Data pre-processing and intrinsic dimension estimation **2**
1.1 Filtering and pre-processing 2
1.2 Intrinsic dimension estimators 2
1.3 Dependence of intrinsic dimension estimations on the sample size and the sub-sampling procedure 3
1.4 Definition of a normalized ID-score 4

2 A comparison of intrinsic dimension estimators for scRNA-seq data **4**
2.1 Tested ID estimators 4
2.2 How the presence of multiple manifolds with heterogeneous dimensionality affects ID estimators 6
2.3 ID across scales: from local to global 7
2.4 Comparison between ID estimators 8

3 The Hopfield model as a toy model of the Waddington landscape **9**

4 The relation between the intrinsic dimension and gene-gene correlations **11**

5 Intrinsic dimension versus alternative differentiation potential correlates **11**

6 ID-score and the inference of developmental trajectories **13**

7 Intrinsic dimension of expression profiles and cellular proliferation **14**

8 Description of the analyzed datasets **15**

9 Supplementary Table **20**

10 Supplementary Figures **21**

1 Data pre-processing and intrinsic dimension estimation

1.1 Filtering and pre-processing

In order to reduce the technical noise due to the sampling procedure involved in RNA sequencing, single-cell expression data are usually normalized [1, 2]. We simply normalized the transcript count x_i^j of gene j in a cell i by the total number of transcripts detected in it $X_i = \sum_{j=1}^{D} x_j^i$. Therefore, each cell is represented by a vector of relative transcript abundances in the expression space. While this procedure has the goal of eliminating the effect of sampling depth on expression variability, it can also remove relevant biological signals. In fact, the heterogeneity of X_i values is also related to physiological features, such as cell size. With the technical development of sequencing protocols, if biases in the number of detected transcripts become negligible, it would be possible to remove the normalization step and account for the effect of the total number of transcripts on data geometry.

We adopted the same cell quality control suggested by the authors of each open access dataset we analyzed. These criteria typically include filters based on the number of detected genes, the total number of transcripts, in addition to exclusion of putative doublets.

We focused only on protein-coding genes following the annotation available on BioMart, a data mining tool accessible via the Ensembl database [3]. The only organism for which a list of protein-coding genes is not available is Hydra. In this case, we used all the transcripts contained in the dataset.

We intentionally minimized the pre-processing procedure to test the robustness of our method, to make it widely applicable and to avoid the introduction of arbitrary data transformations such as complex normalizations, data imputation techniques or non-linear data projections [4]. The hypothesis is that relevant global geometrical properties and trends of the data can be extracted from essentially raw data and could instead be confounded by complex pre-processing procedures.

However, our analysis should not be dramatically affected by standard pipelines, and it could even benefit from data corrections able to attenuate confounding factors such as batch effects [5]. In fact, Supplementary Figure 9 shows an example of how the temporal trend of the ID-score reported in Fig.1E changes when a standard feature selection technique is applied. Specifically, we selected only the highly variable genes following one of the most popular gene selection strategies [6]. The estimation of the ID-score is robust with respect to this choice, and the variability between different sub-samplings (error bars) is reduced.

1.2 Intrinsic dimension estimators

We considered different ID estimators. Among the class of projective or PCA-based methods, we used three different statistical properties of the covariance matrix, all related to the data ID: the number of principal components d_{PCA} required to explain a given percentage of the total data variance (Supplementary Eq. 5); the

entropy of the normalized covariance eigenvalues H_{PCA} (Supplementary Eq. 6) and the complementary of their Gini coefficient G_{PCA} (Supplementary Eq. 7).

In the class of geometric/fractal methods, we selected the TWO-NN estimator [7] and the FCI estimator [8], respectively relying on a local and a multiscale approach. The results reported in the figures of the main text are based on TWO-NN. However, the reported trends are generally very robust to the estimator choice (Supplementary Figs. 13-16). A more detailed comparative analysis and a precise mathematical description of the estimators are reported in the following section 2 of the Supplementary Information.

All ID estimators have a specific dependency on the sample size and on the dimension of the embedding space, i.e., the number of cells and genes in the count matrix (Supplementary Fig. 1). To remove the dependency on the sample size, we randomly sub-sampled a number of cells corresponding to 75% of the least represented cell group (with an upper bound of 5000 cells for computational reasons) and measured the intrinsic dimension with different estimators over 10 independent sub-samplings. The mean and the standard deviation of these values are reported as circles and error bars in the figures. Analogously, we considered the same number of protein-coding genes for all cell sub-populations that have to be compared. The exact number of cells and genes used are dataset-specific and are reported in Supplementary Tab. 1. Since the absolute ID value depends on non-biological variables such as the sample size, we decided to define a scaled ID-score, whose values are in the $[0-1]$ range, to be compared with the potency level (Supplementary Eq. 1).

1.3 Dependence of intrinsic dimension estimations on the sample size and the sub-sampling procedure

As discussed in the main text, the estimated values of intrinsic dimension depend on the sample size in the under-sampled regime. Supplementary Fig. 1A reports this dependence for the TWO-NN estimator applied to two simple synthetic datasets. These datasets are obtained by sampling from a 50-dimensional and a 80-dimensional hypercube, respectively, embedded in a 1000-dimensional space. The true ID value is underestimated across several order of magnitudes of the sample size because of the curse of dimensionality [9].

In the case of real datasets, in particular of scRNA-seq count matrices, even if we do not know the true ID, we can observe the relationship between the estimated ID and the number of data points (Supplementary Fig. 1B). The trend of the curves suggest that we are far from saturation and thus the estimated values still crucially depend on the number of cells available. Fortunately, the primary goal of our analysis is not the exact estimation of the ID. We are mainly interested in the evolution of this quantity during development and differentiation, and in ranking cell populations in terms of potency. To make these ID comparisons meaningful we need to compare the ID of groups with the same number of cells. To this aim, we measure the ID on random subsets of fixed size extracted from each cell cluster. With this procedure we obtained Figures 1-4, where the average ID values and their standard deviations across independent sub-samplings are reported. To determine the size of

each sub-sample, we identify the least represented cell cluster and consider the 75% of its number of cells (see column 3 of Supplementary Tab. 1). For computational reasons, we imposed an upper bound of 5000 cells to the size of the sub-samplings. The number of sub-samplings is set to 10 for every trend we report. Clearly, the variability introduced by the sub-sampling procedure is affected by the initial size of each cluster. The less populated clusters will often exhibit the lowest variability as measured with the standard deviation. The absolute ID values measured with the TWO-NN method, although influenced by the undersampling, are significantly lower ($\sim 10^1$) than the number of genes present in the original library ($\sim 10^4$).

1.4 Definition of a normalized ID-score

Since the absolute ID values have a technical dependency on the sample size, we decided to define a re-scaled ID-score and test its correlation with the potency level. Specifically, the measured ID values are normalized in the $[0-1]$ range, by defining

$$ID_{score} = \frac{y - y_{shift}}{y_{scaling} - y_{shift}}, \tag{1}$$

$$y_{shift} = \min\{t - \sigma_t | t \in \text{ID trend}\}, \tag{2}$$

$$y_{scaling} = \max\{t + \sigma_t | t \in \text{ID trend}\}. \tag{3}$$

The y value corresponds to the average estimated ID (with the chosen estimator) over the random sub-samplings. For every dataset, we subtract the minimum ID value y_{shift} and we scale the values with the maximum value $y_{scaling}$. The values of y_{shift} and $y_{scaling}$ are computed taking into account the variability across sub-samplings. Therefore, the ID-scores in the paper figures are constrained in the interval $[0-1]$ with the error bars included. The values of these parameters for each dataset are reported in Supplementary Tab. 1.

2 A comparison of intrinsic dimension estimators for scRNA-seq data

2.1 Tested ID estimators

Among the plethora of intrinsic dimension estimators, we can first distinguish between global estimators and local estimators. Local estimators focus on data neighborhoods of a given scale, while global estimators look at the geometry of the whole dataset. An alternative distinction can be made between projective, and fractal or nearest neighbor-based estimators.

We tested estimators belonging to different classes and relying on different assumptions. Specifically, TWO-NN is local and belongs to the class of nearest-neighbors methods. FCI is a multiscale approach and can be classified as a fractal method. Finally, PCA-based ID estimators are global quantities based on linear projections.

- The TWO-NN algorithm has been proposed in [7] and is based on the computation of the typical distances among neighboring points (cells) in the data space. In particular, the method reconstructs the distribution of the distance ratios μ. For each data point, the value $\mu = r_2/r_1$ is the ratio between its distance to its second (r_2) and first (r_1) neighbor.

  Assuming local data density homogeneity and considering the sampling process as a Poisson point process, Elena Facco et al. demonstrated that μ follows a Pareto distribution $f(\mu)$:

  $$f(\mu) = d\mu^{-d-1}, \quad \mu = \frac{r_2}{r_1}, \tag{4}$$

  with an exponent defined by the intrinsic dimension d. The ID is inferred from a fit on the curve $\{\log \mu, -\log(1 - F(\mu)\}$, where F is the cumulative function of f.

  To make the fit robust to outliers we discard the highest values of μ and consider only the remaining 90% of values, as suggested by the authors of [7].

  Due to the normalization (see Supplementary section 1.1), our data points have non-integer coordinate values. The metric we used to compute the distances is the euclidean distance. The Manhattan distance could be more appropriate if one decide to use directly the integer transcript counts.

- The Full Correlation Integral (FCI) [10] is a fractal method that has been recently proposed as a development of the classic Grassberger and Procaccia method [11] for undersampled datasets.

- PCA-based observables. Given N cells, D genes and a counts matrix $\boldsymbol{X_{N\times D}}$, the eigenvalues $\boldsymbol{\lambda} = (\lambda_1, \lambda_2, \ldots, \lambda_m)$ (where $m = \min\{N, D\}$) of the covariance matrix $\boldsymbol{C_{D\times D}}$ specify the contribution to the total variance of the data given by each eigenvector or principal component. The relative magnitude of these values contains information about the intrinsic dimension of the dataset. We can count how many components d_{PCA} are needed to retain a certain percentage V_{th} of the total data variance V_0, and use this value as a proxy for the ID. In other words, we can define:

  $$d_{PCA} = \min_{d\in\mathbb{Z}^+} \left\{ d : \sum_{i=1}^{d} \tilde{\lambda}_i \geq V_{\text{th}} \right\}, \quad V_0 = \sum_{i=1}^{D} \lambda_i, \quad \tilde{\lambda}_i = \frac{\lambda_i}{V_0}, \tag{5}$$

  With this definition, the absolute ID value depends on an arbitrary parameter V_{th}, but again we can define a normalized ID-score.

  Alternatively, we can introduce two other methods of evaluating the non-uniformity of the set of eigenvalues $\boldsymbol{\lambda}$, with the general idea that the level of heterogeneity in the eigenvalues increases as the ID decreases. Specifically, we defined two measures based on the Shannon entropy (H_{PCA}) and on the Gini index (G_{PCA}) following the expressions:

$$H_{PCA} = -\frac{1}{V_0}\sum_{i=1}^{m}\lambda_i \log \lambda_i, \tag{6}$$

$$G_{PCA} = 1 - \frac{1}{2m^2\bar{\lambda}}\sum_{i=1}^{m}\sum_{j=1}^{m}|\lambda_i - \lambda_j|, \quad \bar{\lambda} = \frac{1}{m}\sum_{i=1}^{m}\lambda_i. \tag{7}$$

These two quantities mainly differ in the weight attributed to small eigenvalues. The entropy-based index of Supplementary Eq. (6) is generally more sensitive to them.

PCA-like methods are linear methods. Therefore, if the true data manifold has a curvature, ID estimations based on linear projections will systematically overestimate the ID. Finally, PCA-based method are reliable only if the number of samples is greater than $d\log(d)$, with d being the (usually unkown) intrinsic dimension [8].

2.2 How the presence of multiple manifolds with heterogeneous dimensionality affects ID estimators

Single-cell data are characterized by strong heterogeneity having both biological and technical origin [2]. A large source of biological variability in the datasets we analyzed is the presence of multiple cell types (Supplementary Fig. 5). Since each cell type is characterized by a specific gene expression program and set of regulations, the geometry of expression profiles of cells belonging to different cell types could be radically different and characterized by different intrinsic dimensions. In other words, the points of our dataset could be partitioned on different manifolds with specific geometrical properties and dimensions, embedded in the gene expression space. However, most ID estimators implicitly assume the presence of a single true ID that have to be estimated [12]

In this section, we analyze what is the effect of having a composition of manifolds of different dimensionality on the ID values that can be obtained with TWO-NN and PCA-based estimators. In particular, we consider the illustrative case of points distributed over two manifolds of different dimensions using synthetic (Supplementary Figs. 3A,B) and real data (Supplementary Figs. 3C,D). The main result is that all estimators do not report an average or intermediate value between the true IDs, but are generally biased towards low dimensional values.

- **PCA-based methods.** If a dataset is composed of two clusters that are well-separated in the gene expression space, the direction of separation could be detected as the first principal component, as it captures a large variance. Consequently, the covariance matrix will have a very large first eigenvalue, which can lead to a biased estimation of the ID towards low values (see Supplementary Eqs. 5 6 7), regardless of the actual ID of the two clusters.

  This is clear in the examples in Supplementary Fig. 3A and C. The first principal component is the direction along which the two clusters of red and

blue points separate. More quantitatively, if we consider separately erythroid and primitive streak cells (Supplementary Fig. 3C), we estimate respectively d_{PCA}=3 and d_{PCA}=381 (with threshold V_{th}=0.9). On the other hand, if we consider the dataset as a whole, we estimate d_{PCA}=2. The pronounced separation of expression profiles of these two cell types is probably due to the fact that erythroid cells do not have nuclei and organelles. The presence of such cell "outliers" can bias the PCA-based estimators towards low ID values.

- **TWO-NN.** In the TWO-NN method, points lying on low-ID manifolds have a greater impact on the overall estimated ID. The method relies on the distribution of the distance ratios μ (Supplementary Eq. (4)), where the right tail (i.e., large values) is dominated by points sampled from low-ID manifolds. Intuitively, in high dimensions, neighboring data points are more likely to be at similar distances.
  An example of this effect is reported in Supplementary Fig. 3B and D. The two plots show the cumulative distribution $F(\mu)$, which is used to estimate the ID by TWO-NN. This distribution is evaluated either on the whole dataset or separately on the two manifolds (two hypercubes in B or two different cell types in D). The global distribution is much closer to the one corresponding to the low-dimensional part of the dataset. Therefore, in the presence of manifolds of different dimensions, TWO-NN estimates on the whole dataset are strongly biased towards low dimensions. On the other hand, thanks to its local nature, TWO-NN exhibits better tolerance for isolated outliers compared to PCA-based estimators.

These considerations can be extended to the case of many cell types. The ID can naturally drop if the number of cell types in the dataset increases and the cell types are associated to heterogeneous IDs. In fact, if we build artificial datasets by randomly assemble data relative to different cell types, we can observe an average decrease of the ID with the number of cell types (Supplementary Figs. 6A,B)

In several datasets related to development, the number of cell types naturally increases with time following differentiation lineages. Therefore, the ID decrease we observe in time can be due to cell differentiation, but also to the growth in the number of cell types. In fact, the ID-score is generally correlated with this quantity in developmental datasets (Supplementary Fig. 6C). Precisely to separate the two contributions and establish the ID-score as a measure of potency, we also analyzed single differentiation lineages following a single cell type as it differentiates.

2.3 ID across scales: from local to global

As discussed in the previous section, the heterogeneity of the data manifolds can bias the different estimators in different ways. For example, single outliers can bias global PCA-based estimators towards low dimensional values. On the other hand, fractal and local methods such as TWO-NN are more robust to outliers but still sensitive to dimensional heterogeneity. To better understand the role of the scale (number of neighbor points considered) on the ID estimation in presence of

heterogeneous datasets, we introduce a local PCA estimator $Ld_{PCA,l}$. The parameter l sets the observation scale through the number of neighbor points used to evaluate the covariance matrix. With this local covariance matrix, we can apply the d_{PCA} method (Supplementary Eq. 5) to estimate the ID. By changing the scale l, we can highlight and better understand some critical aspects of the ID estimators in presence of heterogeneous datasets. In general, considering too few data neighbors can lead to strong undersampling and noisy covariance estimates, while using too many neighbours may cause a drop of the ID, due to the influence of outliers or to the presence of multiple cell types (Supplementary Fig. 3 and 6).

Supplementary Fig. 7 shows in detail how the scale affects the ID estimations on one illustrative dataset from mouse gastrulation [13]. We randomly selected 50 cells in the dataset and their first l neighbours (fixing the scale). We then computed the intrinsic dimension of these 50 cell groups and examined their distribution. At very small scales, the d_{PCA} increases with l, just because we are in an extremely undersampled regime and the ID increases with the number of points considered, as in Supplementary Fig. 1. At intermediate scales we start to observe the separation of cell types, and thus a broad and multimodal distribution of IDs. At these scales, the neighborhood of a cell is typically composed by cells of the same cell type. Here, around $l = 411$, we can appreciate that cells of the caudal epiblast and mesoderm show a high intrinsic dimension, while the erythroids have a smaller ID with respect to haematoendothelial and blood progenitors. This ranking largely reflects the known level of specialization of those cell types.

At large enough scales, the data neighbors contains cells of different cell types and the value of local $Ld_{PCA,l}$ progressively collapse on the global estimator $d_{PCA,l}$ as expected.

Since in the dataset are present different cell types, the globally estimated ID converges to a value biased towards the low dimensional values, as explained in Supplementary Section 2.2.

2.4 Comparison between ID estimators

This section reports the results of the tests of robustness of our main results with respect to the choice of the ID estimator. In particular, we evaluated the correlation between the ID-score defined with several estimators (Supplementary section 2.1) on each dataset analyzed for the Figures 1, 2, 3 and 4. We also introduce another local estimator $LG_{PCA,l}$, which corresponds to a local version of G_{PCA} (Supplementary Eq. (7)), with a parameter l setting the observation scale, i.e., the number of neighbor data points used to evaluate the covariance matrix. We explored several values of l to understand effects related to the observation scale.

Supplementary Figures 13, 14, 15 and 16 show how the ID-based rankings obtained with different estimators are generally remarkably conserved. Some disagreement is present in only few cases relative to temporal trends, that are characterized by a strong heterogeneity in cell type composition (Supplementary Fig. 5). As discussed above, some estimators are quite sensitive to the number and heterogeneity of data manifolds, thus explaining the reduced robustness of results in these cases.

Supplementary Fig. 14B constitutes an illustrative example of this effect. In this case, we verified by projecting the data with PCA, the presence of a small number of outliers well separated from the bulk of the dataset in the gene expression space. Therefore, global estimators based on PCA are expected to strongly underestimate the ID. As a proof, we can observe the agreement between local estimators (TWO-NN and $LG_{PCA,120}$), while more global estimators (FCI or PCA-based observables) lead to different rankings. However, when we get rid of this heterogeneity by manually removing those outliers the agreement between estimators can be restored. In fact, when we apply our analysis to single cell types, the estimations based on different methods are robustly correlated (Supplementary Fig. 15 and 16) .

3 The Hopfield model as a toy model of the Waddington landscape

As discussed in the main text, we selected the Hopfield model [14] to preliminary test our hypothesis because it shares some key features with the Waddington landscape metaphor. Specifically, the Hopfield model is characterized by an energy landscape with attractors, which correspond to stored memories in the original model. These state attractors can be associated to the typical expression profiles of various fully differentiated cell types in our analogy. In contrast, a stem or multipotent cell is not constrained within a single attractor. Instead, the differentiation process progressively aligns its expression profile with that of a committed cell type. Accordingly, temperature in the Hopfield model can be used as a parameter to regulate the level of "differentiation". By decreasing the temperature, we can mimic the differentiation process, akin to increasing the number of geometric constraints on the configurations. The key question is whether the intrinsic dimension can quantitatively capture this "differentiation process" in the Hopfield model. In other words, if the intrinsic dimension of an ensemble of spin configurations has a specific increasing trend with temperature.

The correspondence between the Hopfield model and the Waddington landscape has been often invoked, and used to build computational tools for transcriptomic data [15, 16, 17, 18, 19, 20, 21]. However, we do not claim that the Hopfield model provides a direct, accurate, and quantitative description of the epigenetic process of cell differentiation. Instead, we aim to test the hypothesis that the ID can well capture the progressive increase of geometrical constraints as the temperature decreases in a model with a complex landscape where all the relevant parameters are under control.

To make the analogy more precise, a spin configuration corresponds to a single-cell transcriptomic profile, although the expression levels are discretized to -1 or 1 in the classic Hopfield formulation. The number of spins in our model (D) is chosen to be comparable to the number of genes typically present in RNA-seq dataset analysis ($D \simeq 1\,000$). The number of trajectories we simulate (N) corresponds to the number of cells in a scRNA-seq experiment. For example, $N = 1\,500$ in the experiments reported in Supplementary Fig. 2. We tried to reproduce the high-

dimensional nature of our datasets in the analogous Hopfield model to more reliably check our expectations about the intrinsic dimension, since intuition often fails in high-dimensional settings.

The P attractors ξ^μ (the memories of the Hopfield model) are set as random spin configurations. These memories represent the "archetypal" transcriptional profiles of fully differentiated cell types. In the toy model these patterns are random configurations with no particular structure or mutual relations, as it is instead probably the case for transcriptional profiles.

The weights defining the couplings between spins are set by the rule: $W_{ij} = \frac{1}{P}\sum_{\mu=1}^{P}\xi_i^\mu\xi_j^\mu$. The energy of a state S is defined by $H = -\sum_{j<i=0}^{D} W_{ij}S_iS_j$.

The Hopfield model has a critical value α_c for the capacity $\alpha = P/D$ [22]. When the number of stored patterns is below the critical capacity $\alpha_c = 0.138$ (and the temperature is below 1), the stored patterns are stable attractors in the energy landscape. We are interested in this regime as an analogous of the Waddington landscape. Given our parameter setting, we have $\alpha = \frac{P}{D} \simeq 0.01$, so we are indeed in the correct region $\alpha < \alpha_c$. We used Markov Chain Montecarlo (MCMC) to simulate the system evolution, introducing a temperature that, in our analogy, should set the "potency".

We estimated the ID trend with temperature using a PCA-based estimator (Supplementary Eq. 6) . The simulations were repeated 10 times with different random initialization, and we report in Supplementary Fig. 2A the mean values and the standard deviations (shaded area) of the estimated IDs. Despite the fluctuations, the average ID has precisely the expected trend: it decreases if we "freeze" the system. This is not simply due to the occupation of different basins of attraction (Supplementary section 2.2), but rather a consequence of the increased geometrical constraints. In fact, the trend is the same if we consider one single stored memory and we initialize all trajectories in this minimum, thus exploring a single basin of attraction at different temperatures (Supplementary Fig. 2B). Using the Hopfield model, we can also test how the ID estimation depends on the number of minima. As the number of attractors increases, PCA-based estimators are expected to report a significant decrease in the ID, as the directions between attractors capture a large portion of the variance. To test this, we ran simulations of the Hopfield model at a fixed temperature but with a variable number of stored patterns (ranging from 1 to 20, as shown in Supplementary Fig. 2C). This setup is designed to mimic the effect of increasing the number of cell types. We observed that the ID initially decreases with the number of attractors, which aligns with our intuition. As the number of memories increases further, the ID appears to slightly recover and stabilize. Ideally, with a very large number of attractors, the ID would be constrained only by the sample size.

It is important to note that, unlike in transcriptomic data in presence of multiple cell types, the heterogeneity among the basins of attraction in this model is likely much smaller. The different memories stored in the Hopfield model are equivalent random patterns, whereas transcriptomic profiles likely exhibit meaningful structure and substantial heterogeneity in the intrinsic dimension of the manifold corresponding to different cell types. These factors can easily explain the quantitative difference

in the behavior of the ID with respect to the number of attractors in the model and in real transcriptomic data.

4 The relation between the intrinsic dimension and gene-gene correlations

The observation that the ID decreases with differentiation is coherent with the Waddington landscape picture and with the idea that the expression profiles of differentiated and specialized cells are highly regulated. Gene regulation should, in turn, induce observable correlations between gene expression levels. Equivalently, from a landscape perspective, the presence of geometrical constraints should induce correlation between features. Indeed, the increase in heterogeneity of the eigenvalues of the covariance matrix, which we observe using PCA-based estimators during differentiation, also generally correspond to an increased level of feature covariance.

While the intrinsic dimension well recapitulates the global geometrical properties of the dataset, we can anyway analyze the gene-gene correlation structure during development as a consistency check. To this aim, we can define the network of gene-gene correlation and study its evolution. Specifically, for each developmental stage, we first selected the first 3000 highly variable genes - to reduce the computational complexity- using *Scanpy* [23] standard pre-processing function, setting *seurat_v3* as flavor. We then constructed a gene-gene co-expression network by weighting each link with the Pearson correlation coefficient ρ. Links were pruned if $|\rho|$ was smaller than a specified threshold, for example 0.4, 0.5 or 0.6. The number of non-isolated genes (genes with at least one link with a weight exceeding the threshold) can be used as a proxy for the global level of correlation. Supplementary Fig. 4 shows how this quantity increases during mouse gastrulation [13], suggesting a gradual increase in gene-gene correlation with development and supporting the idea that differentiation is accompanied by tighter gene regulation.

However, the number of sufficiently correlated gene pairs only captures a specific aspect of the data statistics during differentiation. For example, all genes are considered equally important, regardless of their average expression level or variance. In contrast, the intrinsic dimension appears to be a more general and straightforward measure of the global geometrical properties of the data, which can be correlated with biological properties such as cell potency.

5 Intrinsic dimension versus alternative differentiation potential correlates

This section analyzes two previously proposed correlates of cell potency that, like intrinsic dimension, are based on simple statistical/geometrical properties of expression profiles without requiring prior biological knowledge.

A scRNA-seq experiment provides a transcript count matrix x^i_j, with $i = 1, 2, ..., N$ (N is the number of cells) and $j = 1, 2, ..., D$ (D is the number of genes). x^i is the

vector representing the expression profile of the i-th cell. Gulati et al. [24] observed that the number of detected genes (i.e., the genes with at least one detected transcript) decreases with cell differentiation (Figure 1 of ref. [24]). They called this quantity Transcriptional Diversity (TD), and it is defined for every cell i as $TD_i = \sum_{j=1}^{D} \mathbf{1}(x_j^i \neq 0)$, i.e. the number of counts in the cell that are not zero. A possible rationale behind the TD trend with potency is that stem cell are less regulated and thus have more diverse expression profiles with respect to strongly regulated differentiated cells.

Another quantity, derived from statistical physics, that have been shown to correlate with cell potency in some datasets is the entropy of expression profiles [25, 26]. The entropy of single-cell expression profiles is defined as $TS_i = \sum_{j=1}^{D} \widetilde{x_j^i} \log(\widetilde{x_j^i})$, where the number of reads assigned to each gene j in cell i is normalized by the total number of reads assigned to the cell, i.e. $\widetilde{x_j^i} = x_j^i / \sum_{j=1}^{D} x_j^i$.

Transcriptional diversity and expression entropy form the basis of much more sophisticated tools, such as CytoTRACE [24] and StemID [26], which also incorporate additional information into their pipelines. However, our aim is not to benchmark these tools in detail, but rather to analyze and test the easily computable (and interpretable) statistical properties on which they are built as proxies of cell potency, and to compare them with intrinsic dimension.

While these two quantities (transcriptional diversity and entropy) sometimes correlate with cell potency in the dataset we analyzed, the correlation is less systematic compared to the ID-score. Supplementary Fig. 12 shows two examples of comparisons between ID-score, TD and TS in recovering differentiation potentials in mouse gastrulation [13] and pancreatic endocrinogenesis [27]. While the ID-score can reproduce the known hierarchies, in these examples both TD and TS produce incorrect rankings. Sometimes the expected hierarchy is even reversed.

It is important to notice that both the transcriptional diversity and the expression entropy are properties of single cells, while the ID-score is a score for a group of cells. In fact, Supplementary Fig. 12 compares the distributions of TD and TS as box plots, with the ID measured over different sub-samplings. This difference can make the ID a more robust measure of global properties of the dataset, less sensitive to noise from stochastic gene expression and from technical origins. The downside is that the ID-score cannot attribute a potency score to every single cell, but requires a preliminary cell grouping or clustering.

Moreover, entropy and transcriptional diversity are strongly dependent on number of detected genes. Transcriptional diversity is directly defined by this quantity, while the maximum value of entropy depends on this quantity. However, the number of detected genes is highly affected by the sequencing depth and by the sampling process involved in RNA sequencing. A global geometric measure, such as the ID, should be less sensitive to data sparsity due to sampling. These two factors could explain the higher robustness of the ID-score as a proxy for potency that we observed in the analyzed datasets.

6 ID-score and the inference of developmental trajectories

This section provides the details of the analysis for the inference of developmental trajectory and pseudotime, reported in Fig. 5 and Supplementary Fig. 8.

We considered the dataset on human gastrulation [28] as an illustrative example. The first step in trajectory reconstruction is data preprocessing. Here, we simply selected human protein-coding genes [3] and normalized the count matrix (scanpy.pp.normalize_total). The data was already been filtered using quality controls by the authors. Subsequently, we applied log1p transformation (scanpy.pp.log1p), we found the first 2000 highly variable genes (scanpy.pp.highly_variable_genes, n_top_genes=2000), and proceeded with neighbors identification (scanpy.pp.neighbors, n_neighbors=15, n_pcs=15,method='umap'). Finally, we computed the diffusion map [29] using the function scanpy.tl.diffmap (adata, n_comps=15). The embedding of cells in the space of diffusion components is solely used to identify cell neighborhoods by binning/clustering. However, the ID measurement were performed using the original (normalized) gene expression matrix.

Fig. 5A and Supplementary Fig. 8A,C display the two-dimensional representations derived from diffusion components 1 and 2. In trajectory reconstruction and pseudotime analysis, it is standard practice to select a "root" cell within this low-dimensional structure. This cell is then assigned "pseudotime = 0", and the pseudotime of other cells is quantified using their distance from this root [30].

A careful examination of the diffusion map in Fig. 5A reveals a branching structure with three prominent vertices. This representation naturally suggests placing the root at one of these three diffusion map vertices. To select the root cell, we employ the ID-score. Each cell is assigned an ID based on its nearest neighbors, with distances computed in the diffusion map embedding. For the analysis presented in Fig. 5, the ID for each cell was determined using its 70 nearest neighbors in the two-dimensional embedding space (geometrically, this is analogous to smoothed clustering through axis binning in Fig. 5A). Specifically, we performed 40 subsampling iterations, each time randomly selecting 50 cells from the 70-cell neighborhood, and assigned each cell the average ID value across these subsamples. For the analysis shown in Supplementary Fig. 8, the same pipeline was applied. However, in this case, neighbors were identified using all 15 diffusion components. Each cell ID was then computed from 40 subsamples of 20 cells drawn from its 35 nearest neighbors. After computing the ID for each cell, we apply scaling and normalization to obtain the standard ID-score in the [0, 1] range (Supplementary equation (1)), which is visualized by color in both the diffusion map (Fig. 5A, Supplementary Fig. 8A) and the UMAP embedding (Fig. 5B, Supplementary Fig. 8B).

Within the embedding of the first two diffusion components of Fig. 5A, high ID-scoring cells densely populate one of the previously mentioned vertices, specifically the one with the lowest value along diffusion component 2, which corresponds to the epiblast. Indeed these cells are supposed to have the highest developmental potency. Similarly, in Supplementary Fig. 8, high ID-scores are also associated with the epiblast, as illustrated in Supplementary Fig. 8C,G. Accordingly, we selected the

cell with the maximum ID-score as the root (marked with a red cross) for subsequent pseudotime ordering. The coloring of Fig. 5D and Supplementary Fig. 8F shows the consistency between pseudotime and ID-score, further supported by the scatterplot in Fig. 5C and Supplementary Fig. 8E, confirming a strong Pearson correlation between these two quantities.

Instead of calculating the ID individually for each cell and its neighbors, a more computationally efficient alternative is to cluster cells and compute the ID-score at the cluster level. For this dataset, the authors provide predefined clusters along with cell type annotations. Up to this point, we intentionally ignored cell type information to demonstrate the utility of the ID-score in the absence of prior biological assumptions. In fact, in poorly characterized systems or for rare cell types, the ID-score can offer valuable information about relative differentiation levels, thereby aiding in cell type annotation. Another computational strategy could be examining how the cell types are placed within the diffusion space and evaluating the ID-score per cluster. As discussed previously, the epiblast exhibits the highest ID-score, corresponding to the same vertex previously identified. From this vertex, two distinct branches emerge: one comprising endoderm cells, the other composed of various mesoderm maturation stages.

7 Intrinsic dimension of expression profiles and cellular proliferation

The level of cell proliferation is often correlated with cell potency [31, 32], therefore the relation between ID and cell potency could also be influenced by a dependence of the ID on cell proliferation. From single-cell RNA-seq data, transcriptional signatures can be defined to estimate the level of cell proliferation.

The first transcriptional proxy we considered is based on the procedure of ref. [27]. The authors calculate a gene-informed score for cell cycle phase S and G2/M. Given a cell, if both scores are negative, they classify it as non-cycling, otherwise as cycling. We consider the percentage of cycling cells within a population as an estimate of the proliferation level. We adopted this approach to all the considered dataset. More specifically, we inferred the cell-cycle phase using the function *tl.score_genes_cell_cycle* of *Scanpy* [33] with default parameters and calculated the percentage of cycling cells for different populations. To distinguish between the S and G2 / M phases, the aforementioned function needs a list of genes and we used the set of 97 cell-cycle related genes defined by ref. [34].

Another possible transcriptional proxy of cell proliferation is the pseudo-proliferation index introduced by ref. [35]. This index is defined using the mean expression of 20 genes that are known in the literature to be associated with proliferation.

The Supplementary Figures 10 and 11 report the result of the analysis on the role of cell proliferation and are discussed in the Discussion section of the main text. To summarize, while the ID-score is often correlated with the proxies we considered of the proliferation rate, there are several exceptions. In general, the ID seems to consistently capture the potency hierarchies across datasets. Moreover, we did not

find any consistent dependence of the intrinsic dimension on the cell-cycle stage of cells at the same level of potency (i.e., of the same cell type).

8 Description of the analyzed datasets

The freely-available scRNA-seq datasets that we considered for our analyses are largely from the Gene Expression Omnibus repository and rely on experimental protocols using Unique Molecular Identifiers (UMIs). We considered datasets, listed below, that refers to both whole embryos or single organs or tissues as a function of time. In most of the datasets, cells are annotated by the authors depending on both the developmental stage and the cell type.

Mouse Cell Atlas

Mouse cell atlas [36, 37] is a dataset obtained via Microwell-seq. Hundreds of single cells have been sequenced covering all of the major mouse organs and the cell types have been identified using a clustering procedure. The analyzed time-stages are: fetal (embryonic day E14.5), neonatal, ten days, three weeks, and adult (6-10 weeks).

We considered the 8498 protein-coding genes that have been sampled in every cell of every organ.

Mouse Haematopoietic stem/progenitor cells

In the homeostasis of several adult tissues, multipotent progenitor cells continuously differentiate into specialized cell types in a continuous self-regulating process of regeneration and renewal. For example, all cellular blood components are derived from hematopoietic stem cells during the hematopoiesis.

In [38], 44.802 single-cell transcriptomes are reported, covering the hematopoietic stem/progenitor (HSPC) compartment from mouse bone marrow. The sequencing protocols are Smart-Seq2 and 10x Chromium.

To obtain a comprehensive view of hematopoiesis, the cells are sorted in two broad gates: LK, capturing HSPCs, and Lin$^-$Sca-1$^+$c-Kit$^+$ (LSK), a subset of the LK gate enriched for more immature progenitors. The sorting gate to isolate LSK and LK cells was based on c-Kit and Sca-1 surface expression for droplet-based scRNA-seq.

Mouse Gastrulation and early organogenesis

Ref. [13] investigates the dynamic process of cellular diversification during gastrulation and early organogenesis of mice that occurs in the 48 hours spanning from embryonic day (E) 6.5 to E8.5. Profiles from whole mouse embryos were collected at six-hour intervals between E6.5 to E8.5. In total, 116.312 single-cell transcriptomic profiles were clustered and annotated, identifying 37 major cell populations. ScRNA-seq libraries were generated using the 10x Genomics Chromium system (v.1 chemistry) and samples were sequenced on an Illumina Hi-Seq 2500 platform.

In this dataset, the information about the specific biological samples is provided. Therefore, to reconstruct the temporal ID trend of Fig. 1B, we restricted our analysis to cells coming from the same sample (for each developmental stage), in order to reduce possible batch effects. We could not do the same distinction for Fig. 3B due to the limited number of cells.

Mouse pancreatic endocrinogenesis

The formation of pancreatic endocrine cells occurs in two distinct stages during rodent embryonic development. The first stage occurs between E9.0 and E12.5, while the second one between E12.5 and E15.5, during which the majority of endocrine cell types are formed. With the aim of resolving the molecular changes during endocrinogenesis, high-throughput scRNA-seq analysis of 38.000 pancreatic epithelial cells during the secondary transition (E12.5, E13.5, E14.5, and E15.5) was performed [27]. More specifically, four scRNA-seq experiments were performed using 10X Genomics technology. Unsupervised graph-based clustering revealed eight major cell clusters including multipotent pancreatic progenitors (MPCs), tip, trunk, acinar, ductal, EPs, Fev^{high}, and endocrine cells. Cell clusters were annotated using the expression of well-known marker genes and the lineage relationships between these cell types were fully reconstructed [27].

Mouse corticogenesis

To build a comprehensive single-cell transcriptional and epigenetic atlas of the developing mammalian cerebral cortex, the authors of [39] performed scRNA-seq experiments over the entire period of corticogenesis: E10.5 and E11.5 (symmetrically dividing neuroepithelial cells); E12.5 and E13.5 (birth date of layer 6 and 5 excitatory neurons); E14.5 to E17.5 (birth date of layer 4 and 2/3 excitatory neurons); and E18.5, postnatal day (P) 1 and P4 (gliogenesis). They report 98.047 scRNA-seq profiles, which include all known cell types of the developing cerebral cortex.

They used a pseudotime approach based on diffusion (URD) [40], to generate a branching tree of trajectories based on the transcriptional similarity of pseudotime ordered cells. The root consists of early progenitors belonging to E10.5, the tips come from P4 and correspond to terminal cell types.

Here, we focus on the embryonic time stages of corticogenesis, from E10.5 to E18.5. We also investigate how the ID-score recapitulates the differentiation trajectories of excitatory neurons in the two progressive stages: from apical to intermediate progenitors, until the state of excitatory neurons.

Mouse embryoids

In Ref. [41], they applied tiny sci-RNA-seq3 to profile 285.640 single cell transcriptomes of multiple individual 'ETiX' mouse embryoids assembled from embryonic stem cells, trophoblast stem cells and inducible extraembryonic endoderm stem cells. The data concern the day 6 and day 8 of development. Amadei et al. also studied scRNA-seq data of natural mouse embryos at E7.5, E8, E8.5, E8.75. and E9.

The aim of their work consisted in showing that ETiX mouse embryoids can firstly develop into gastrulating embryoids, and secondly into neurulating embryoids. In conclusion, they found out that the ETiX embryoids recapitulate the development of whole natural mouse embryos in uterus up to day 8.5 post-fertilization.

In our study, we only used data from embryoids to compare the value of ID at day 6 and 8 (Fig. 2E).

Zebrafish embryogenesis - Wagner

In the study [42], over 92.000 cells from zebrafish embryos were subjected to inDrops single-cell RNA sequencing throughout the initial day of development. Employing a graph-based methodology together with analysis of known marker genes, the researchers delineated a cell-state landscape elucidating axis patterning, germ layer formation, and organogenesis. The data come from 7 different developmental stages (4 hours post-fertilization, 6 hpf, 8 hpf, 10 hpf, 14 hpf, 18 hpf and 24 hpf).

Zebrafish embryogenesis - Farrell

In [40], Farrell et al. studied single-cell transcriptomes of 38.731 cells obtained with Drop-seq during early zebrafish embryogenesis at a high temporal resolution, spanning 12 stages from the onset of zygotic transcription through early somitogenesis. To identify the transcriptional trajectories in the data they developed a simulated diffusion-based computational approach (URD), which identified the trajectories describing the specification of 25 cell types in the form of a branching tree, where the the root of the tree correspond to a multipotent cell type, while terminally differentiated cell types constitutes the tips.

Zebrafish neurogenesis

A 2020 study by Raj et al. [43] analyzed the gene expression of over 220,000 individual cells isolated in zebrafish brains, using the 10X Chromium scRNA-seq platform. These cells originated from 12 distinct developmental stages, spanning from embryo to larva. Analyses of known and novel marker genes revealed about 800 clusters and allowed to characterize the transition from progenitors to neurons and, more in general, the molecular mechanisms underlying vertebrate neurogenesis.

Zebrafish embryogenesis - Farnsworth

In [44], the study was conducted on 44.102 cells coming from 6 different experiments and concerning the development of zebrafish embryos. From single-cell RNA sequencing data, Farnsworth et al. computationally reconstructed 220 clusters and annotated them by analysing the expression of some marker genes, identified through ZFIN database (Zebrafish Information Network). ScRNA-seq libraries were generated using the 10x Genomics Chromium platform (v.2 chemistry) and samples were sequenced on either an Illumina Hi-Seq or an Illumina Next-seq.

In Fig. 3D, we compare the ID of three classes of cell types (being represented at least by 180 cells) that take part in the genesis of retinal neurons and photoreceptors. These classes are retinal progenitors (RetProgAlla, RetProg1a, RetProg0a, RetProg0b, RetProgAllb, RetProgAllc), differentiating retinal cells (RetDiff2, RetDiff25a, RetDiffAll, RetDiff25b, RetDiff25c, RetDiff25d, RetDiff25e) and retinal neurons (RetNeuron25, RetPR - retinal photoreceptors).

Hydra turnover

The cnidarian polyp Hydra undergoes continual self-renewal and is capable of whole-body regeneration from a small piece of tissue. The stem cell populations, morphological cell types and lineage relationships are well characterized. In [45], they sequenced 24.985 single-cell transcriptomes and identified the molecular signatures of a broad spectrum of cell states, from stem cells and progenitors to terminally differentiated cells, building differentiation trajectories for all cell lineages. In our work, for the two epithelial layers (endoderm and ectoderm) and the interstitial lineage we compared the ID of progenitors and differentiated cell clusters, selecting only sufficiently represented cell types having more than 330 cells.

In Fig. 4A, the points refer to cell types belonging to the interstitial layer and are ordered in the following way: stem/progenitor cells, neuronal cells (nc) progenitors, nc and gland cells (gc) progenitors, nematoblast (nb) 1, nb 2, differentiated nematocyte, ectodermal neuron (n_ec) 1, n_ec 2, male germline, granular mucous gland cells, spumous mucous gland cells 2, zymogen gland cells 1. Moving on to endodermal epithelilal cells, in Fig. 4B, points are respectively referred to: stem cells (SC) 1, SC2, SC3, cells from head, cells from foot, cells from tentacles. Finally, the order chosen for ectoderm in Fig. 4C is: SC1, SC2, differentiated nematocyte, cells from head, basal disk, multiplets of battery cells 2, nematoblasts (suspected phagocytosis doublet).

C. elegans embryogenesis

In [46], the authors sequenced the transcriptomes of single cells from C. elegans embryos with the 10X Genomics platform. They assayed loosely synchronized embryos enriched for preterminal cells as well as embryos that had been allowed to develop for ∼300, ∼400, and ∼500 min after the first cleavage of the fertilized egg. After the quality control, they estimated the embryo stage of the 86.024 single cells by comparing their expression profile with a high-resolution whole-embryo RNA-seq time series [47].

Human gastrulation

To validate models of human gastrulation, Tyser, Mahammadov, et al. [28] sought to compare the transcriptome of the human gastrula with those of stage-matched non-human model organisms, specifically the mouse and cynomolgus monkey, as well as a human embryonic stem (ES) cell-based in vitro model.

The authors conducted a detailed analysis of a human embryo at Carnegie Stage (CS) 7 from the HDBR, it was was karyotypically and morphologically normal, representing typical human gastrulation at that stage. To preserve anatomical context during scRNA-seq, the embryo was micro-dissected into three regions: the yolk sac, the rostral embryonic disk, and the caudal embryonic disk. This spatial annotation allowed partial spatial resolution of the resulting transcriptomes. Single-cell mRNA was isolated and amplified using the SMART-seq2 protocol (without unique molecular identifiers, UMIs), and sequencing was performed on an Illumina HiSeq 2500 platform (V4 chemistry).

Following stringent quality control, including tests to exclude maternal contamination and verify normal cell cycling, a library of 1.195 high-quality single-cell transcriptomes was retained. Unsupervised clustering identified 11 distinct cell populations, which were annotated based on anatomical location and established marker genes. These populations included: epiblast, ectoderm (amniotic/embryonic), primitive streak, nascent mesoderm, axial mesoderm, emergent mesoderm, advanced mesoderm, extraembryonic mesoderm, endoderm, haemato-endothelial progenitors (HEPs), and erythroblasts. Using diffusion maps and RNA velocity analysis, the authors identified two major developmental trajectories emerging from the epiblast: one toward mesoderm and the other toward endoderm.

In Fig. 3F we selected the cell types comprising more than 90 cells and we considered only human protein-coding genes (BioMart, Ensembl database [3]).

9 Supplementary Table

Dataset	Number of cells		Number of genes		Normalization	
	Total	sub-sampled	Total	Protein-coding	y_{shift}	$y_{scaling}$
Mouse gastrulation [13]	108839	1530 p.s.<br>467 p.ct.	29452	23727	34<br>14	60<br>46
Mouse Pancreas [27]	36351	3781 p.s.<br>918 p.ct.	27998	16558	28<br>18	50<br>49
Mouse cell atlas [37, 36]	231732	5000 p.s. every organ<br>1478 p.s. single organ	9348	8498	40<br>36	70<br>84
Mouse corticogenesis [39]	77842	781 p.s.<br>4995 p.ct.	27998	16461	31<br>39	106<br>60
Mouse hematopoiesis [38]	44802	5000 p.b.s.	27998	16109	59	78
Mouse embryoids [41]	285640	1464 p.b.s.	49585	17847	54	114
Zebrafish embryogenesis [40]	38279	142 p.s.<br>154 p.u.s.	23974	14081	27<br>18	77<br>58
Zebrafish embryogenesis [44]	44020	136 p.ct.	32520	23911	9	46
Zebrafish embryogenesis [42]	36749	2676 p.s.	30677	14059	37	87
Zebrafish neurogenesis [43]	137706	5000 p.s.	32191	22905	22	60
Elegans embryogenesis [46]	86024	344 p.s.	20222	18000	8	27
Human gastrulation [28]	1195	90 p.ct.	57490	18506	14	40
Hydra turnover [45]	24984	332 p.ct. interstitial<br>344 p.ct. endoderm<br>337 p.ct. ectoderm	36814	NA	8<br>16<br>15	48<br>32<br>26

Supplementary Tab. 1: **Description of the datasets.** The number of cells sub-sampled from each group (p.s.=per stage, p.ct.=per cell type, p.b.s.=per biological sample, p.u.s=per URD segment) is specified for every analyzed dataset (Figs. 1 2 3 4). The total number of detected genes and protein-coding genes (defined by the Ensembl database [3]) is reported. In the last two columns, we indicate the two parameters (Eq. (2) and (3)) used to define the normalized ID-score.

10 Supplementary Figures

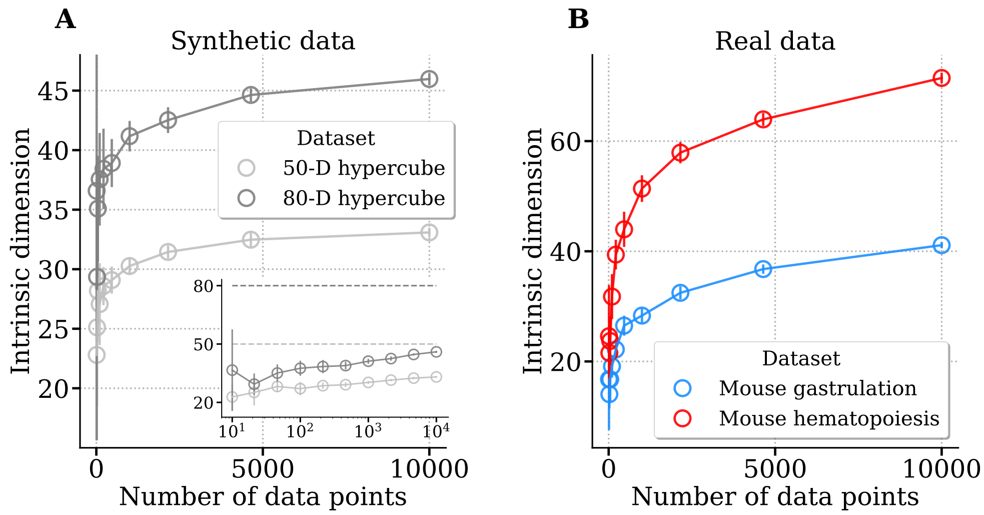

Supplementary Fig. 1: **Relationship between the estimated ID and the sample size. A**) 50-dimensional and a 80-dimensional hypercubes embedded in a 1000-dimensional space are considered. A variable number of points is sampled from these two synthetic datasets. The ID estimations can correctly rank the two manifold dimensions if evaluated at the same sample size. However, the inset shows that the estimated values are far from the true ID (dotted line). **B**) The same analysis is repeated on two real datasets related to mouse gastrulation [13] and mouse hematopoiesis [38] dataset.

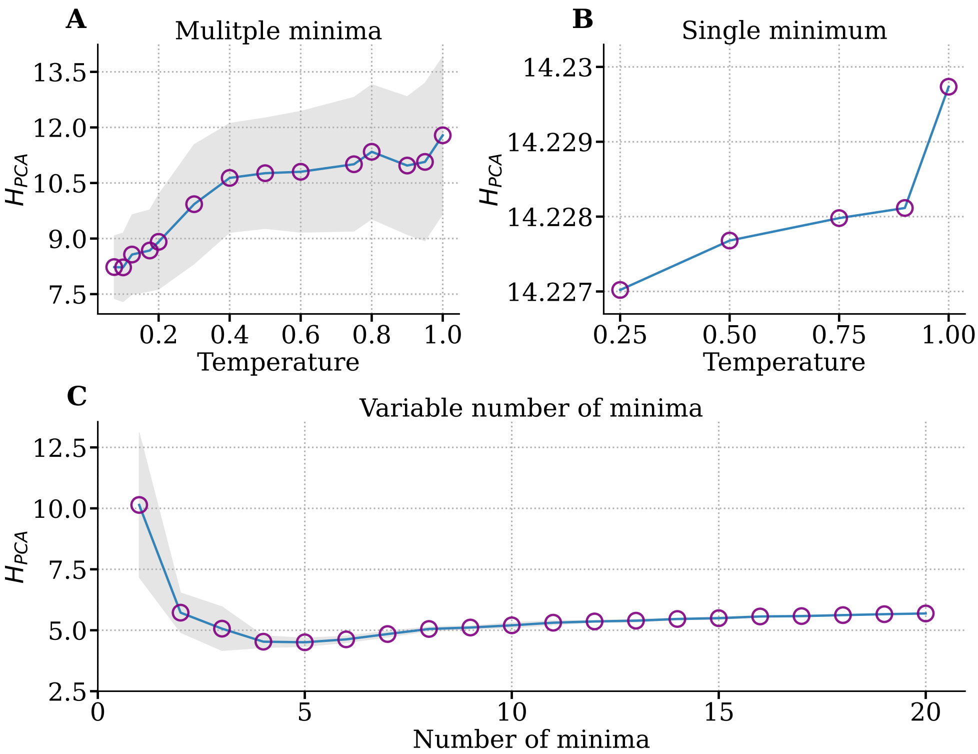

Supplementary Fig. 2: **Intrinsic dimension dependence on temperature and on the number of memories in the Hopfield model. A**) ID values are obtained with the H_{PCA} estimator (Eq. 6). We simulated a fixed number of 1500 randomly initialized trajectories of the Hopfield model with 10 random memory stored at different temperature values. We computed the mean H_{PCA} values over 10 independent simulations and considered a 3-points-window moving average, while the shaded area reports the standard deviations. As hypothesized, the intrinsic dimension decreases as the temperature decreases. **B**) The same simulations are performed with a single random memory stored and with all trajectories initialized in this minimum. Shaded area is not visible because error bars on y values have order magnitude 10^{-15}. **C**) With a fixed temperature of $T = 0.1$, the H_{PCA} is calculated as a function of the number of attractors (memories stored) in the energetic landscape, from 1 to 20. In this case, we consider 250 trajectories and mean values are computed over 10 simulations.

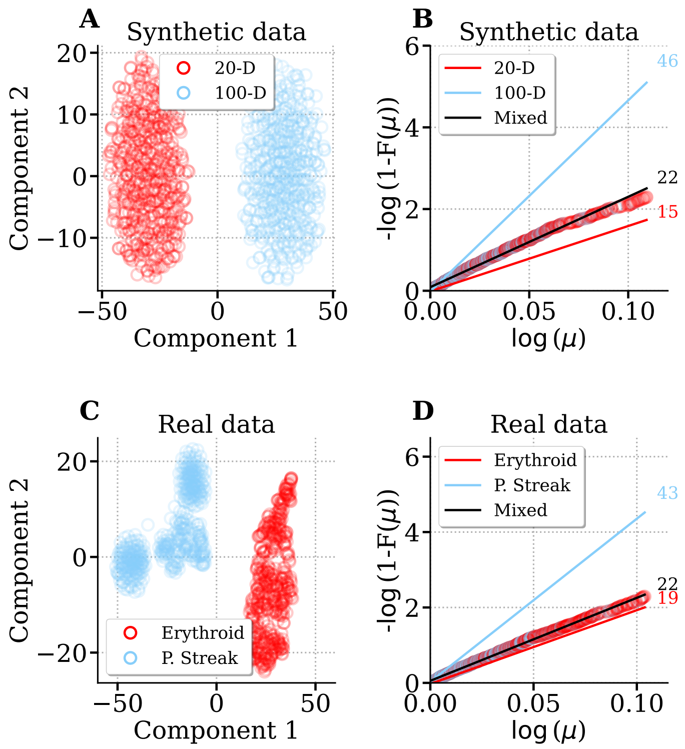

Supplementary Fig. 3: **In the presence of multiple data manifolds ID estimators are biased towards low dimensions.** In the first row: data sampled from a 20 and 100-dimensional hypercube embedded in a 1000-dimensional space, each one represented by 10^3 points. In the second row: data from two cell types (erythroid and primitive streak) from the mouse gastrulation dataset [13], with 10^3 cells per cell type. **A** and **C**) The data projection on the 2D space given by t-SNE shows the presence of two well separated clusters. **B** and **D**) Relationship between μ (introduced in Eq.(4)) and its cumulative distribution $F(\mu)$. The pale blue line and the red one are the fitted lines when single manifolds are considered. The black line is obtained taking the whole dataset. The values reported on the right correspond to the ID estimations based on TWO-NN. Note that we are in the under-sampled regime.

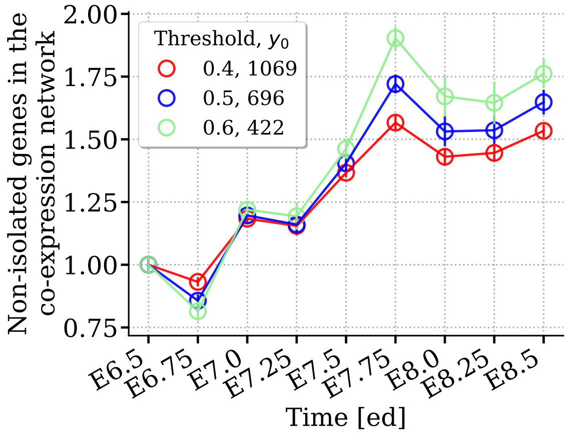

Supplementary Fig. 4: **The correlations among gene expression levels increase during development.** The number of genes in the co-expression network grows with developmental stages in the mouse gastrulation dataset [13]. We report the trend for different threshold values used to construct the network. Values on the y-axis are scaled by the value corresponding to stage E6.5 (y_0), as indicated in the legend.

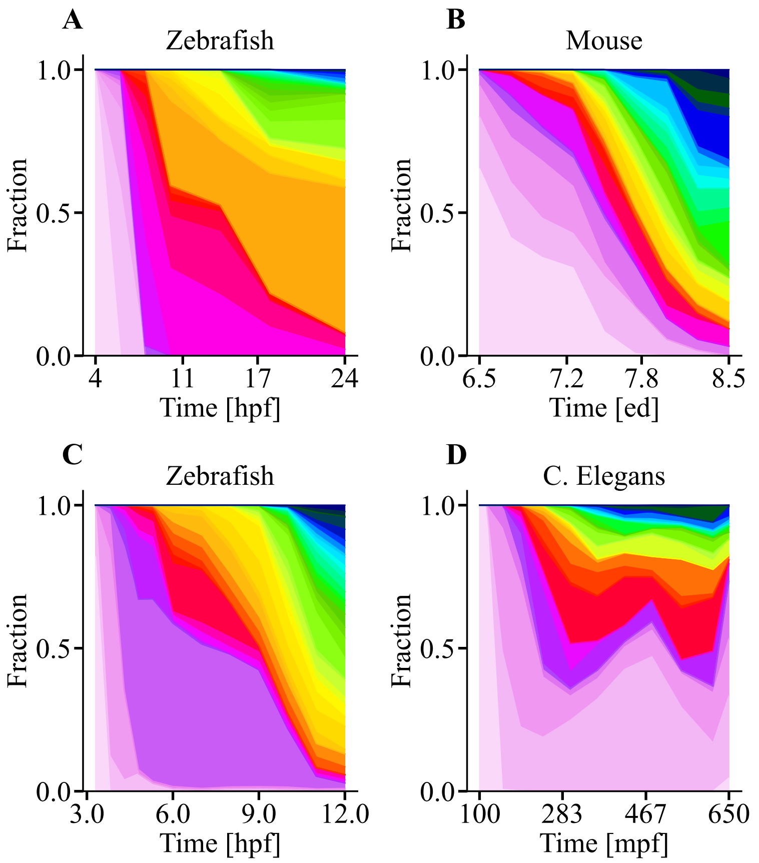

Supplementary Fig. 5: **The number of cell types increases during embryogenesis.** Cell-type proportions during pre-natal development for Zebrafish 4-24 hpf [42] (**A**), Mouse 6.5-8.5 ed [13] (**B**), Zebrafish 3.3-12 hpf [40] (**C**), and C. Elegans 100-650 mpf [46] (**D**). For each animal, each color corresponds to a specific cell type as annotated by the authors. The number of distinct colors increases as a function of developmental time.

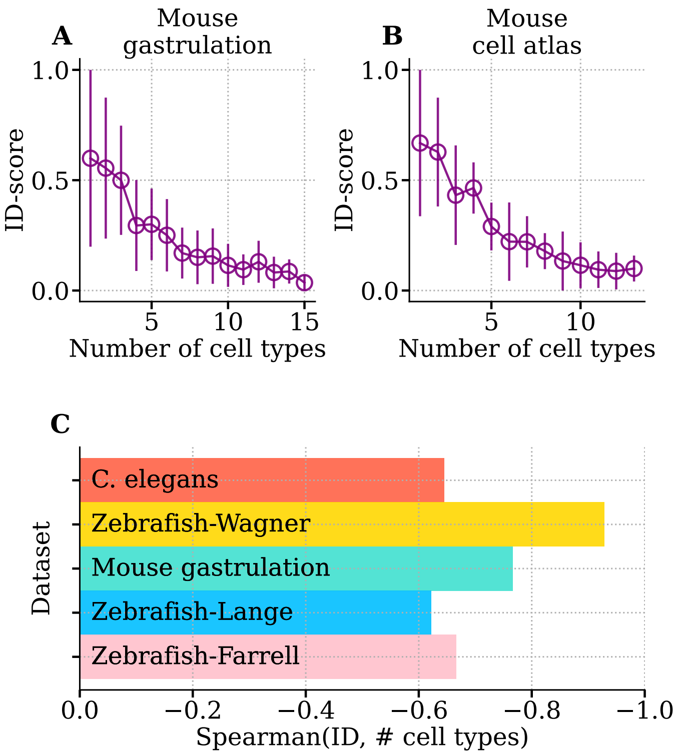

Supplementary Fig. 6: **The ID decreases with the number of cell types independently of their overall level of differentiation.** We report the ID-score (symbol $\pm$ error bar $=$ mean $\pm$ standard deviation) of controlled mixtures of cell types, obtained by randomly assembling cell types from the Mouse gastrulation dataset [13] (**A**) and the Mouse cell atlas [36, 37] (**B**). **C**) Spearman correlation between the number of cell types and the ID-score calculated for different datasets relative to embryonic development.

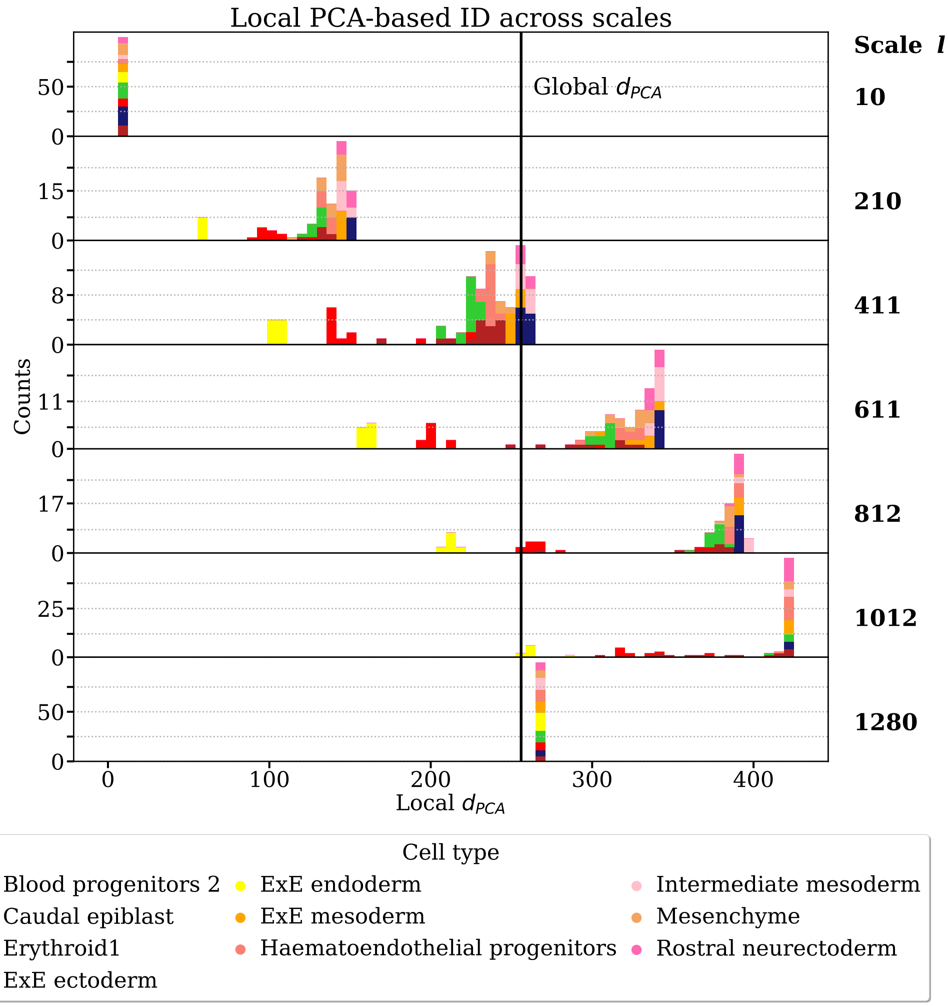

Supplementary Fig. 7: **A local PCA-based estimator reveals cell types heterogeneity across different scales**. The local version of d_{PCA}, i.e., $Ld_{PCA,l}$ is applied at different scales (l) on 1300 cells from embryonic day 8 of mouse gastrulation [13]. at each scale the scale, we measured the local ID on 50 sub-samples of randomly selected cells. The colours encode for the cell type. Each cell type is equally represented by 130 cells. The vertical black line specifies the value of d_{PCA} taking the 1300 cells together.

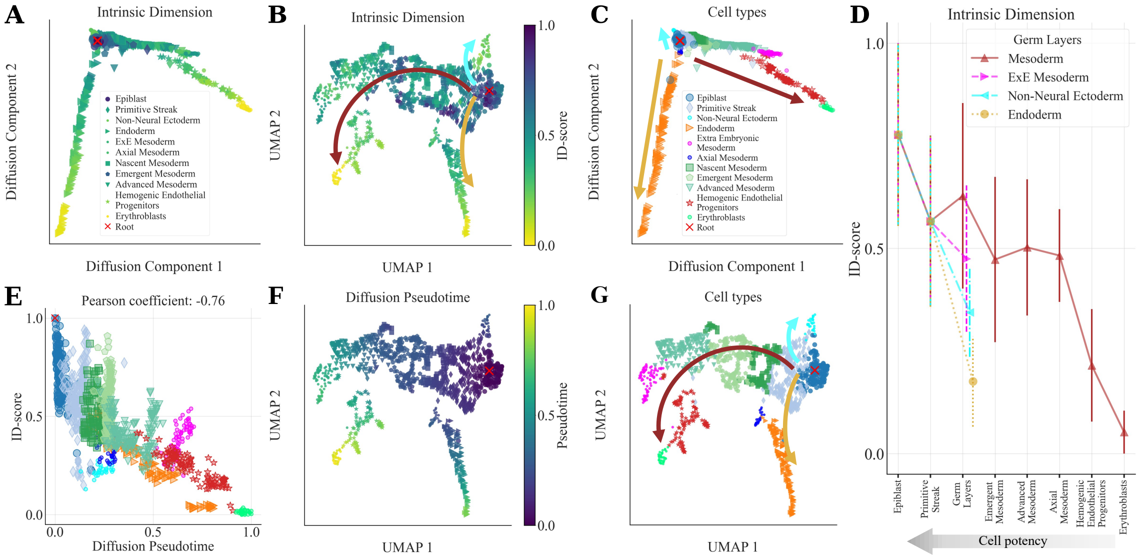

Supplementary Fig. 8: **In-depth analysis of the human gastrulation dataset.** **A)** Diffusion map of all cell types from the human gastrulation dataset [28], with cells colored by the ID-score computed from their 35 nearest neighbors in diffusion component space (15 components used). The cell with the highest ID-score is marked with a red cross and is selected as the "root" for diffusion pseudotime analysis (shown in panels E and F). Legend colors indicate the average ID-score for each cell type and reflect a potency hierarchy consistent with panel D. **B)** UMAP embedding of cells colored by ID-score. Arrows highlight differentiation branches: mesoderm (brown), endoderm (golden), and ectoderm (cyan). **C)** Diffusion map with cells colored by cell type annotations provided by the original authors. Comparison with panel A shows that the root and other high-ID-scoring cells are part of the epiblast population. **D)** Cell type hierarchy along the differentiation trajectories during human gastrulation, as reconstructed in [28]. The ID-score reflects this hierarchy; for each cell type, the ID was computed 300 times using random subsamples of 20 cells. **E)** Scatterplot showing an inverse correlation between ID-score and diffusion pseudotime (Pearson coefficient = −0.76). **F)** UMAP embedding colored by diffusion pseudotime. **G)** UMAP embedding colored by cell type annotation.

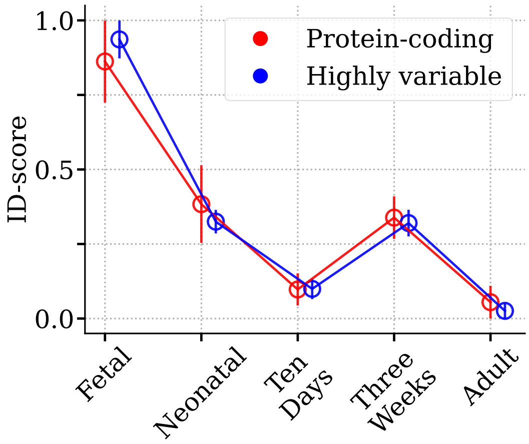

Supplementary Fig. 9: **Robustness of results with respect to feature selection.** ID measured for different temporal stages of the Mouse Cell Atlas dataset [36, 37]. We firstly took into account all 8498 protein-coding genes (red curve), then we restricted the analysis to the first 2000 highly-variable genes detected with *seurat_v3* of *scanpy* (blue curve).

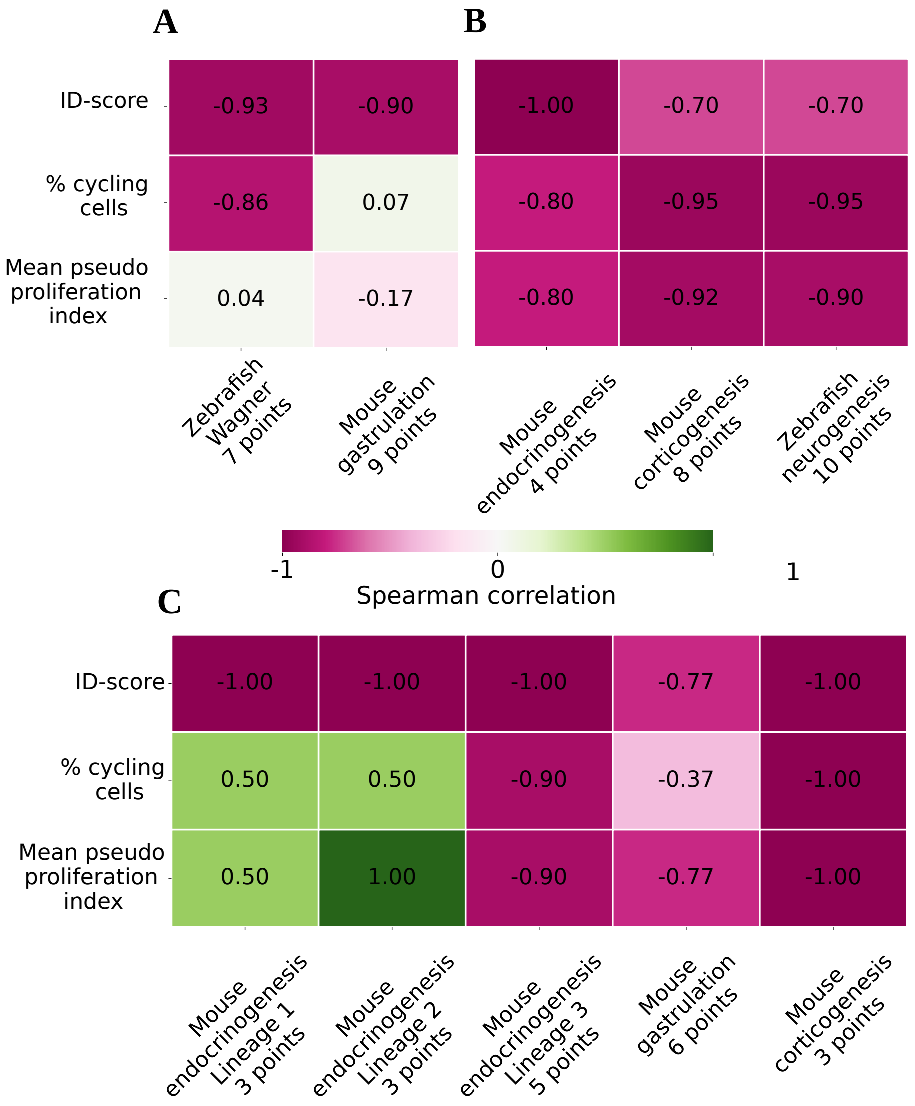

Supplementary Fig. 10: **Correlation between cell potency and cell proliferation indexes.** Spearman correlation is calculated between the developmental time (in A and B) or the potency ranking given by the data annotations (in C) and 3 observables: the ID-score and the two transcriptional proxies of cell proliferation, i.e. the percentage of cycling cells and the mean pseudo-proliferation index, introduced by ref. [35]. The correlation is computed considering the time stages of whole embryo development [42] [13] (**A**) or the development of single organs [27] [39] [43] (**B**). In panel (**C**) the trends are computed with respect to the known potency hierarchies of cell types also used in the main text [27] [13] [39]

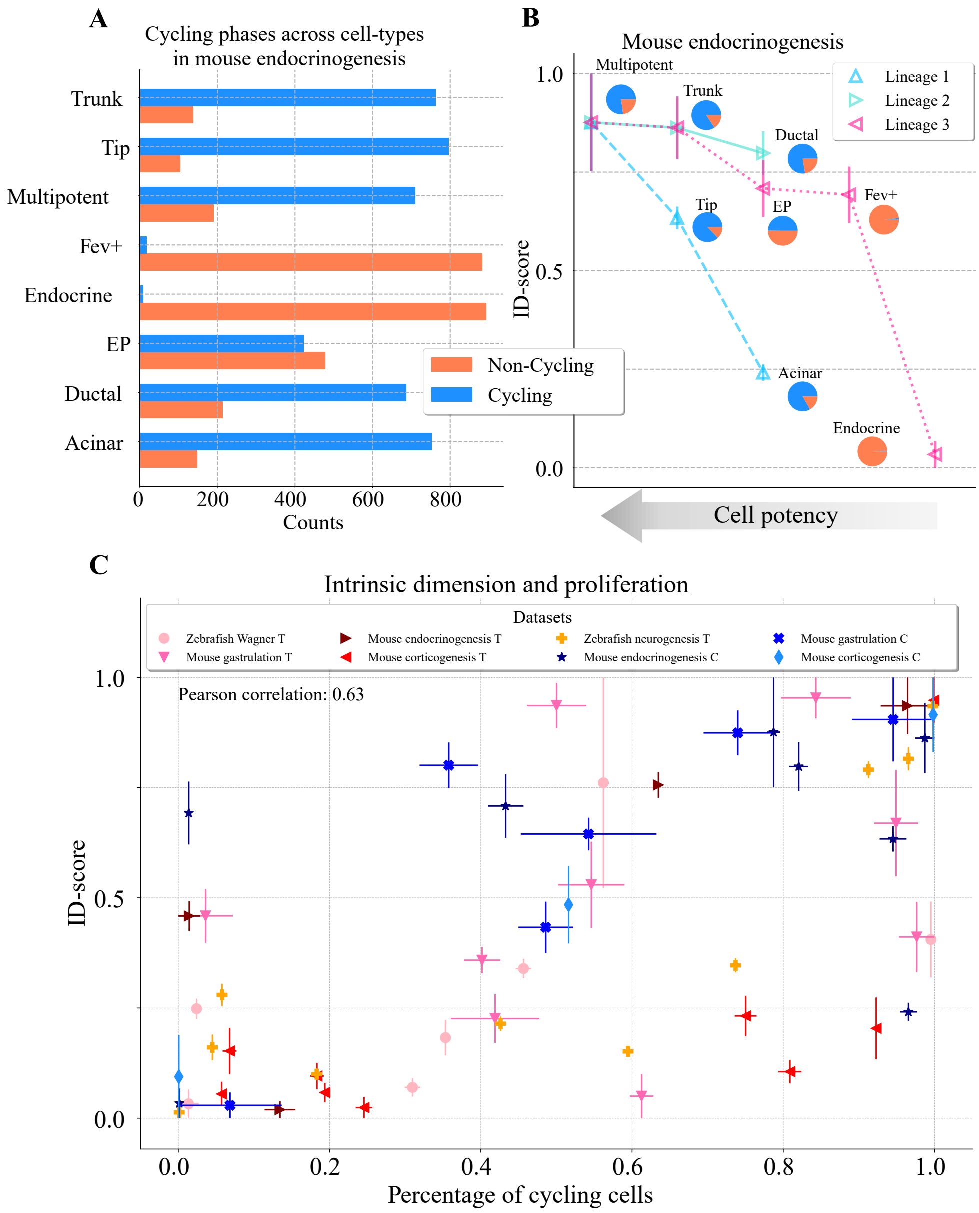

Supplementary Fig. 11: **Relation between intrinsic dimension and percentage of cycling cells across datasets. A**) We report the number of cycling cells over 1000 cells randomly sampled from each cell-type of the mouse pancreatic endocrinogenesis dataset [27]. The inference of cycling cells is based on an expression signature as explained in detail in the Supplementary Section 7. The pie charts in **B**) show the estimated proportion of cycling and non-cycling cells in each cell type as the ID-score decreases with differentiation, as in Fig. [3]. **C**) For every dataset considered in Supplementary Fig. 10, we report the relation between the ID-score and the percentage of cycling cells. We considered datasets where we have expression level as a function of developmental time (denoted with "T" in the legend), and datasets where we can directly order cell types with respect to their potency (denoted with a "C"). We also report the Pearson correlation coefficient (p-value=$2.25 \cdot 10^{-7}$) that indicates a general correlation between the two measures, although many exceptions can be clearly observed.

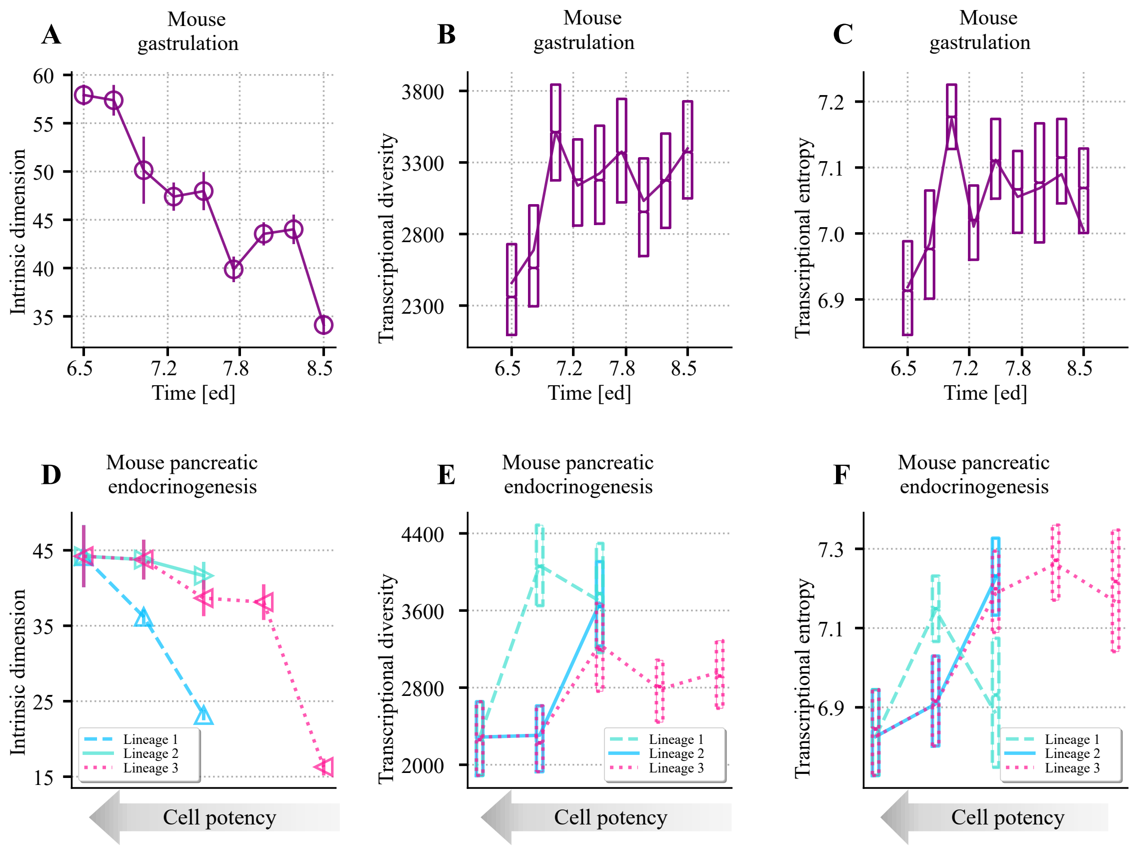

Supplementary Fig. 12: **Comparison between ID, transcriptional diversity and transcriptional entropy.** First row: cells from mouse gastrulation [13] grouped per developmental stage as in Fig. 1B. Second row: the hierarchy of cell types induced by the known differentiation diagram of pancreatic endocrinogenesis [27] (Fig. 3A), composed by lineage 1 (Multipotent-Tip-Acinar), lineage 2 (Multipotent-Trunk-Ductal) and lineage 3 (Multipotent-Trunk-Ep-Fev+-Endocrine). Different lines connect the three lineages as in Fig 3. For each dataset, ID trends are shown (Figures **A** and **D**), together with boxplots resuming transcriptional diversity and transcriptional entropy distributions (Figures **B**,**E** and **C**,**F**).

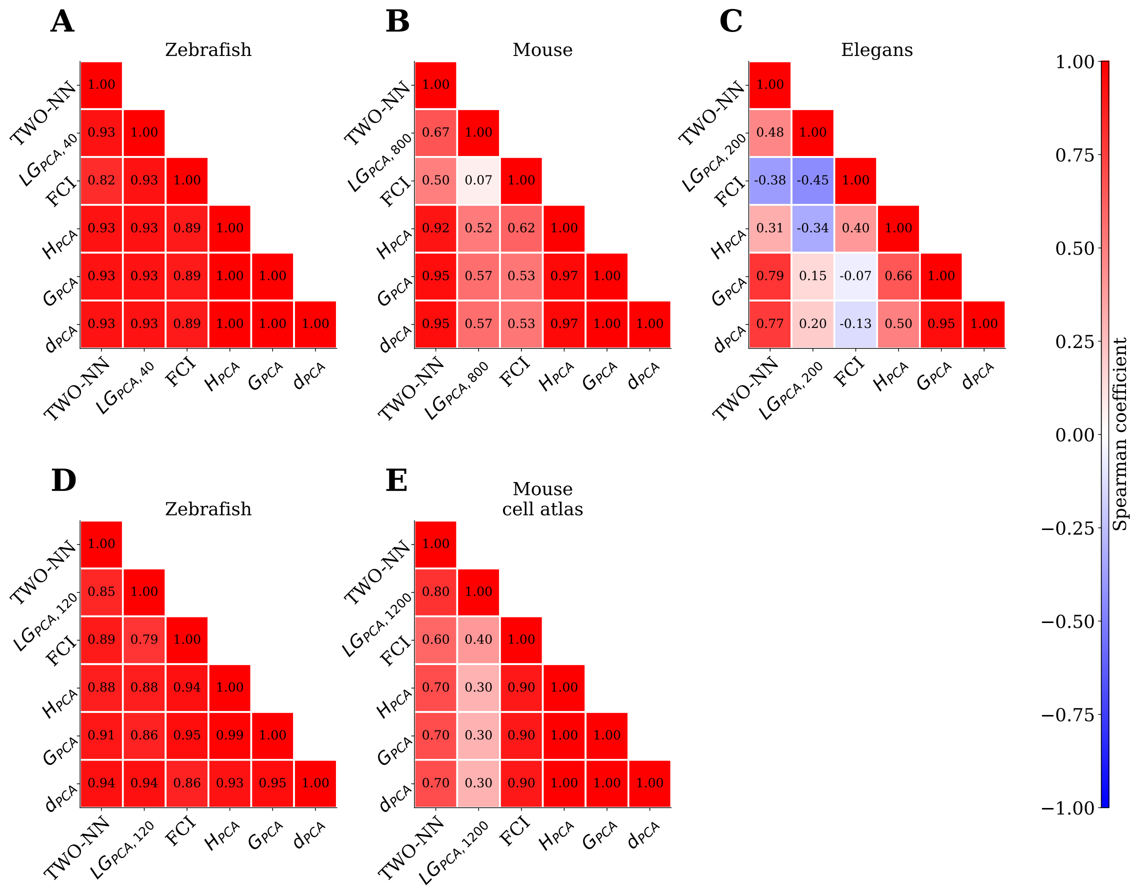

Supplementary Fig. 13: **Correlation between temporal ID trends in whole organisms obtained with different ID estimators.** Each trend showed in Fig. 1 is reproduced with a different ID estimator and the Spearman correlation coefficient is computed for every pair of estimators.

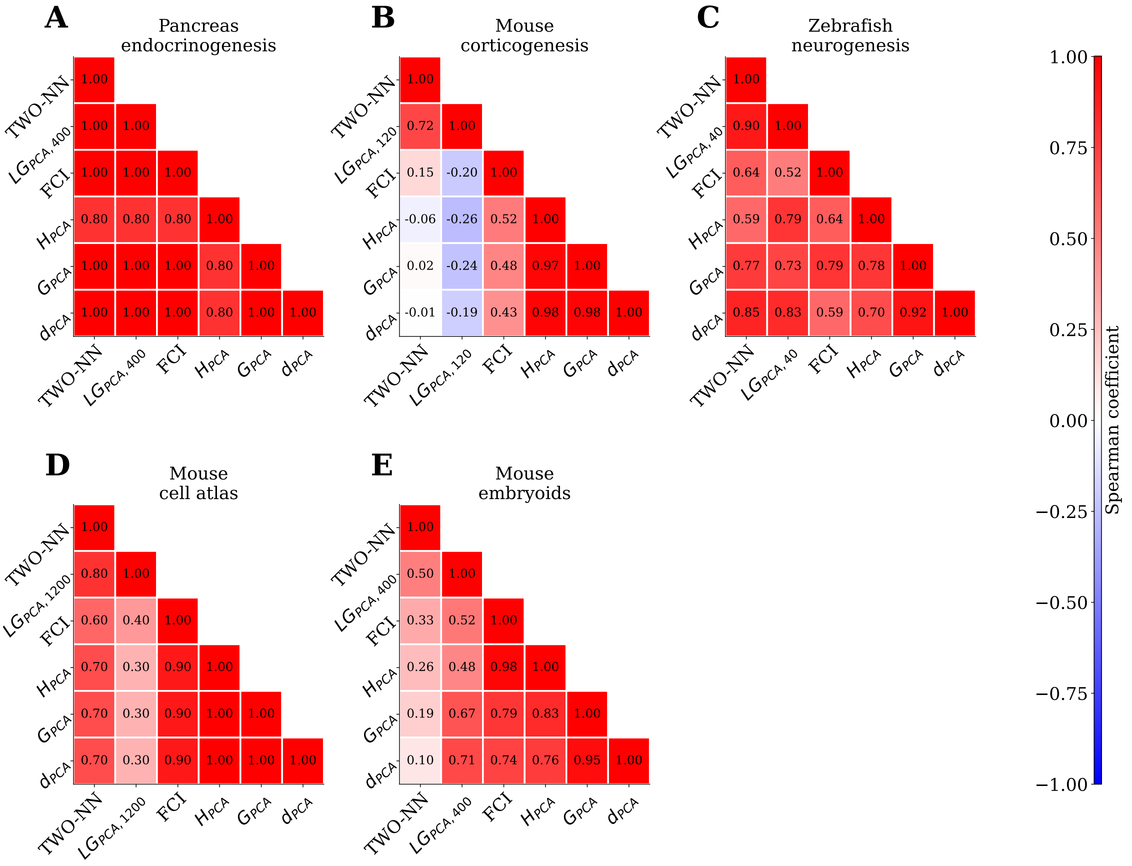

Supplementary Fig. 14: **Correlation between temporal ID trends in single organs obtained with different ID estimators.** Each trend showed in Fig. 2 is reproduced with a different ID estimator and the Spearman correlation coefficient is computed for every pair of estimators.

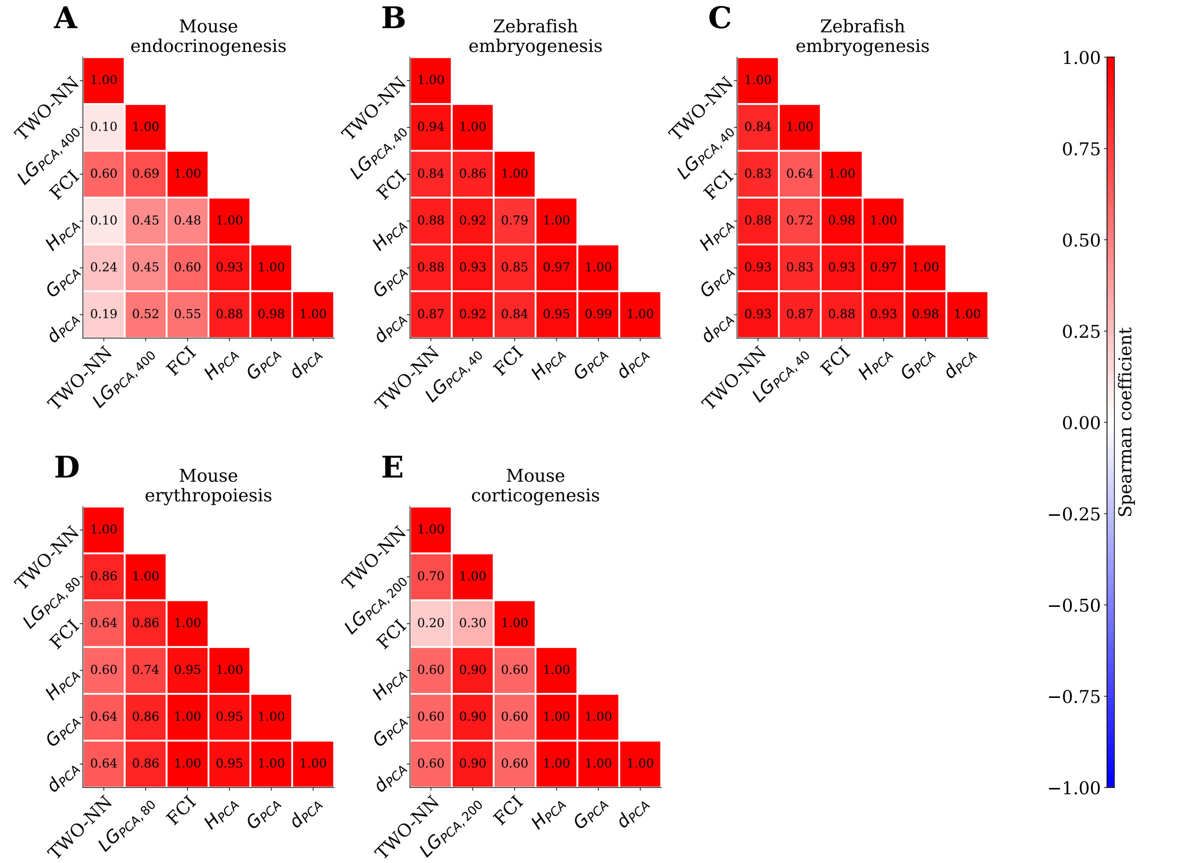

Supplementary Fig. 15: **Correlation between ID values of cell types obtained with different estimators.** Each trend showed in Fig. 3 is reproduced with a different ID estimator and the Spearman correlation coefficient is computed for every pair of estimators.

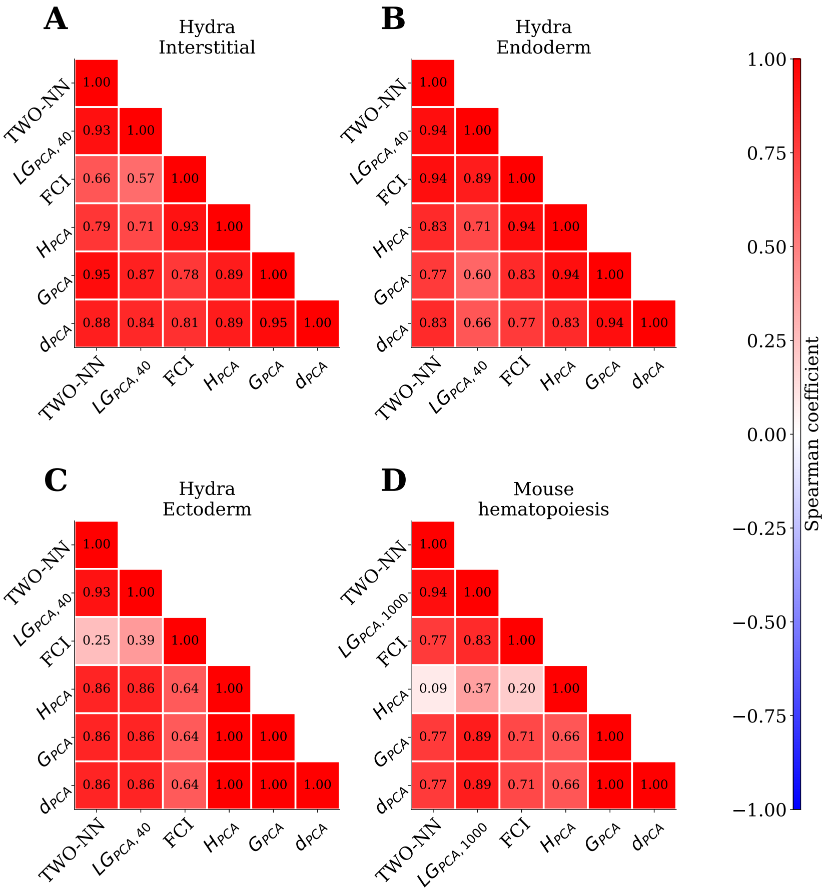

Supplementary Fig. 16: **Correlation between ID values of cell types during tissue renewal obtained with different estimators.** Each trend showed in Fig. 4 is reproduced with a different ID estimator and the Spearman correlation coefficient is computed for every pair of estimators.

References

[1] Abhishek Sarkar and Matthew Stephens. Separating measurement and expression models clarifies confusion in single-cell rna sequencing analysis. *Nature Genetics*, 53(6):770–777, 2021.

[2] Silvia Lazzardi, Filippo Valle, Andrea Mazzolini, Antonio Scialdone, Michele Caselle, and Matteo Osella. Emergent statistical laws in single-cell transcriptomic data. *Physical Review E*, 107(4):044403, 2023.

[3] Fergal J Martin, M Ridwan Amode, Alisha Aneja, Olanrewaju Austine-Orimoloye, Andrey G Azov, If Barnes, Arne Becker, Ruth Bennett, Andrew Berry, Jyothish Bhai, et al. Ensembl 2023. *Nucleic Acids Research*, 51(D1):D933–D941, 2023.

[4] Malte D Luecken and Fabian J Theis. Current best practices in single-cell rna-seq analysis: a tutorial. *Molecular Systems Biology*, 15(6):e8746, 2019.

[5] Laleh Haghverdi, Aaron T. L. Lun, Michael D. Morgan, and John C. Marioni. Batch effects in single-cell rna-sequencing data are corrected by matching mutual nearest neighbors. *Nature Biotechnology*, 36:421–427, 2018.

[6] Rahul Satija, Jeffrey A. Farrell, David G Gennert, Alexander F. Schier, and Aviv Regev. Spatial reconstruction of single-cell gene expression. *Nature Biotechnology*, 33:495 – 502, 2015.

[7] Elena Facco, Maria d'Errico, Alex Rodriguez, and Alessandro Laio. Estimating the intrinsic dimension of datasets by a minimal neighborhood information. *Scientific Reports*, 7(1):12140, 2017.

[8] Vittorio Erba, Marco Gherardi, and Pietro Rotondo. Intrinsic dimension estimation for locally undersampled data. *Scientific Reports*, 9(1):17133, 2019.

[9] Ian Goodfellow, Yoshua Bengio, and Aaron Courville. *Deep learning*. MIT press, 2016.

[10] Vittorio Erba, Marco Gherardi, and Pietro Rotondo. Intrinsic dimension estimation for locally undersampled data. *Nature*, 06 2019.

[11] Peter Grassberger and Itamar Procaccia. Characterization of strange attractors. *Physical Review Letters*, 50(5):346, 1983.

[12] Michele Allegra, Elena Facco, Francesco Denti, Alessandro Laio, and Antonietta Mira. Data segmentation based on the local intrinsic dimension. *Scientific Reports*, 10:16449, 10 2020.

[13] Blanca Pijuan-Sala, Jonathan A Griffiths, Carolina Guibentif, Tom W Hiscock, Wajid Jawaid, Fernando J Calero-Nieto, Carla Mulas, Ximena Ibarra-Soria, Richard CV Tyser, Debbie Lee Lian Ho, et al. A single-cell molecular map of mouse gastrulation and early organogenesis. *Nature*, 566(7745):490–495, 2019.

[14] J J Hopfield. Neural networks and physical systems with emergent collective computational abilities. *Proceedings of the National Academy of Sciences*, 79(8):2554–2558, April 1982.

[15] Stefan R Maetschke and Mark A Ragan. Characterizing cancer subtypes as attractors of hopfield networks. *Bioinformatics*, 30(9):1273–1279, 2014.

[16] Sai Teja Pusuluri, Alex H Lang, Pankaj Mehta, and Horacio E Castillo. Cellular reprogramming dynamics follow a simple 1d reaction coordinate. *Physical Biology*, 15(1):016001, 2017.

[17] Laura Cantini and Michele Caselle. Hope4genes: a hopfield-like class prediction algorithm for transcriptomic data. *Scientific Reports*, 9(1):337, 2019.

[18] Jing Guo and Jie Zheng. Hopland: single-cell pseudotime recovery using continuous hopfield network-based modeling of waddington's epigenetic landscape. *Bioinformatics*, 33(14):i102–i109, 2017.

[19] Maria Yampolskaya, Michael J Herriges, Laertis Ikonomou, Darrell N Kotton, and Pankaj Mehta. sctop: physics-inspired order parameters for cellular identification and visualization. *Development*, 150(21):dev201873, 2023.

[20] Alex H Lang, Hu Li, James J Collins, and Pankaj Mehta. Epigenetic landscapes explain partially reprogrammed cells and identify key reprogramming genes. *PLoS Computational Biology*, 10(8):e1003734, 2014.

[21] Hanshuang Li, Chunshen Long, Yan Hong, Liaofu Luo, and Yongchun Zuo. Characterizing cellular differentiation potency and waddington landscape via energy indicator. *Research*, 6:0118, 2023.

[22] Daniel J Amit, Hanoch Gutfreund, and Haim Sompolinsky. Spin-glass models of neural networks. *Physical Review A*, 32(2):1007, 1985.

[23] F Alexander Wolf, Philipp Angerer, and Fabian J Theis. Scanpy: large-scale single-cell gene expression data analysis. *Genome Biology*, 19:1–5, 2018.

[24] Gunsagar S Gulati, Shaheen S Sikandar, Daniel J Wesche, Anoop Manjunath, Anjan Bharadwaj, Mark J Berger, Francisco Ilagan, Angera H Kuo, Robert W Hsieh, Shang Cai, et al. Single-cell transcriptional diversity is a hallmark of developmental potential. *Science*, 367(6476):405–411, 2020.

[25] Olivier Gandrillon, Mathilde Gaillard, Thibault Espinasse, Nicolas B Garnier, Charles Dussiau, Olivier Kosmider, and Pierre Sujobert. Entropy as a measure of variability and stemness in single-cell transcriptomics. *Current Opinion in Systems Biology*, 27:100348, 2021.

[26] Dominic Grün, Mauro J Muraro, Jean-Charles Boisset, Kay Wiebrands, Anna Lyubimova, Gitanjali Dharmadhikari, Maaike van den Born, Johan Van Es, Erik Jansen, Hans Clevers, et al. De novo prediction of stem cell identity using single-cell transcriptome data. *Cell Stem Cell*, 19(2):266–277, 2016.

[27] Aimée Bastidas-Ponce, Sophie Tritschler, Leander Dony, Katharina Scheibner, Marta Tarquis-Medina, Ciro Salinno, Silvia Schirge, Ingo Burtscher, Anika Böttcher, Fabian J Theis, et al. Comprehensive single cell mrna profiling reveals a detailed roadmap for pancreatic endocrinogenesis. *Development*, 146(12):dev173849, 2019.

[28] Richard CV Tyser, Elmir Mahammadov, Shota Nakanoh, Ludovic Vallier, Antonio Scialdone, and Shankar Srinivas. Single-cell transcriptomic characterization of a gastrulating human embryo. *Nature*, 600(7888):285–289, 2021.

[29] Laleh Haghverdi, Florian Buettner, and Fabian J Theis. Diffusion maps for high-dimensional single-cell analysis of differentiation data. *Bioinformatics*, 31(18):2989–2998, 2015.

[30] Laleh Haghverdi, Maren Büttner, F Alexander Wolf, Florian Buettner, and Fabian J Theis. Diffusion pseudotime robustly reconstructs lineage branching. *Nature Methods*, 13(10):845–848, 2016.

[31] Lijun Liu, Wojciech Michowski, Aleksandra Kolodziejczyk, and Piotr Sicinski. The cell cycle in stem cell proliferation, pluripotency and differentiation. *Nature cell biology*, 21(9):1060–1067, 2019.

[32] Abdenour Soufi and Stephen Dalton. Cycling through developmental decisions: how cell cycle dynamics control pluripotency, differentiation and reprogramming. *Development*, 143(23):4301–4311, 2016.

[33] F. Wolf, Philipp Angerer, and Fabian Theis. Scanpy: Large-scale single-cell gene expression data analysis. *Genome Biology*, 19, 02 2018.

[34] Itay Tirosh, Benjamin Izar, Sanjay M Prakadan, Marc H Wadsworth, Daniel Treacy, John J Trombetta, Asaf Rotem, Christopher Rodman, Christine Lian, George Murphy, et al. Dissecting the multicellular ecosystem of metastatic melanoma by single-cell rna-seq. *Science*, 352(6282):189–196, 2016.

[35] Marie Locard-Paulet, Oana Palasca, and Lars Juhl Jensen. Identifying the genes impacted by cell proliferation in proteomics and transcriptomics studies. *PLOS Computational Biology*, 18(10):e1010604, 2022.

[36] Xiaoping Han, Renying Wang, Yincong Zhou, Lijiang Fei, Huiyu Sun, Shujing Lai, Assieh Saadatpour, Ziming Zhou, Haide Chen, Fang Ye, et al. Mapping the mouse cell atlas by microwell-seq. *Cell*, 172(5):1091–1107, 2018.

[37] Xiaoping Han, Ziming Zhou, Lijiang Fei, Huiyu Sun, Renying Wang, Yao Chen, Haide Chen, Jingjing Wang, Huanna Tang, Wenhao Ge, et al. Construction of a human cell landscape at single-cell level. *Nature*, 581(7808):303–309, 2020.

[38] Joakim S Dahlin, Fiona K Hamey, Blanca Pijuan-Sala, Mairi Shepherd, Winnie WY Lau, Sonia Nestorowa, Caleb Weinreb, Samuel Wolock, Rebecca Hannah, Evangelia Diamanti, et al. A single-cell hematopoietic landscape resolves

8 lineage trajectories and defects in kit mutant mice. *Blood*, 131(21):e1–e11, 2018.

[39] Daniela J Di Bella, Ehsan Habibi, Robert R Stickels, Gabriele Scalia, Juliana Brown, Payman Yadollahpour, Sung Min Yang, Catherine Abbate, Tommaso Biancalani, Evan Z Macosko, et al. Molecular logic of cellular diversification in the mouse cerebral cortex. *Nature*, 595(7868):554–559, 2021.

[40] Jeffrey A Farrell, Yiqun Wang, Samantha J Riesenfeld, Karthik Shekhar, Aviv Regev, and Alexander F Schier. Single-cell reconstruction of developmental trajectories during zebrafish embryogenesis. *Science*, 360(6392):eaar3131, 2018.

[41] Gianluca Amadei, Charlotte E Handford, Chengxiang Qiu, Joachim De Jonghe, Hannah Greenfeld, Martin Tran, Beth K Martin, Dong-Yuan Chen, Alejandro Aguilera-Castrejon, Jacob H Hanna, et al. Embryo model completes gastrulation to neurulation and organogenesis. *Nature*, 610(7930):143–153, 2022.

[42] Daniel E Wagner, Caleb Weinreb, Zach M Collins, James A Briggs, Sean G Megason, and Allon M Klein. Single-cell mapping of gene expression landscapes and lineage in the zebrafish embryo. *Science*, 360(6392):981–987, 2018.

[43] Bushra Raj, Jeffrey Farrell, Jialin Liu, Jakob El Kholtei, Adam Carte, Joaquin Acedo, Lucia Du, Aaron McKenna, Dorde Relić, Jessica Leslie, and Alexander Schier. Emergence of neuronal diversity during vertebrate brain development. *Neuron*, 108, 10 2020.

[44] Dylan Farnsworth, Lauren Saunders, and Adam Miller. A single-cell transcriptome atlas for zebrafish development. *Developmental Biology*, 459, 11 2019.

[45] Stefan Siebert, Jeffrey A Farrell, Jack F Cazet, Yashodara Abeykoon, Abby S Primack, Christine E Schnitzler, and Celina E Juliano. Stem cell differentiation trajectories in hydra resolved at single-cell resolution. *Science*, 365(6451):eaav9314, 2019.

[46] Jonathan S Packer, Qin Zhu, Chau Huynh, Priya Sivaramakrishnan, Elicia Preston, Hannah Dueck, Derek Stefanik, Kai Tan, Cole Trapnell, Junhyong Kim, et al. A lineage-resolved molecular atlas of c. elegans embryogenesis at single-cell resolution. *Science*, 365(6459):eaax1971, 2019.

[47] Tamar Hashimshony, Martin Feder, Michal Levin, Brian K Hall, and Itai Yanai. Spatiotemporal transcriptomics reveals the evolutionary history of the endoderm germ layer. *Nature*, 519(7542):219–222, 2015.
